# Substrate secretion by different EHEC secretion systems during their interaction with epithelial cells

**DOI:** 10.1080/21505594.2026.2634461

**Published:** 2026-02-19

**Authors:** Landy Zambrano-Arguello, Jaime Vazquez-Lopez, Fernando Navarro-Garcia

**Affiliations:** Department of Cell Biology, Centro de Investigación y de Estudios Avanzados (Cinvestav), Mexico City, Mexico

**Keywords:** *Escherichia coli*, secretion systems, protein secretion, EHEC-epithelial cells interaction, proteomics, virulence factors, bacterial effectors

## Abstract

Enterohemorrhagic *Escherichia coli* (EHEC) causes severe foodborne illness in humans. EHEC harbors five types of secretion systems (SSs) and unlike the type three secretion system (T3SS), the other SSs are less explored. Some substrates secreted by these SSs have been described; however, how these SSs collectively participate in EHEC-epithelial cell interaction during infection remains unknown. Here, we optimized protein secretion by four of the EHEC SSs in the absence and presence of epithelial cells, since the T6SS is not expressed *in vitro*. The secretion of substrates through the EHEC SSs followed a hierarchical pattern when bacteria encountered epithelial cells. Epithelial cells increase the protein secretion by EHEC and mutants in SSs (Western-blot and proteomics). Remarkably, mutants in one particular SS affect protein secretion by other SSs. Analysis of the EHEC secretome in the absence/presence of cells vs different SS mutants showed that epithelial cells increase the abundance of specific substrates; the lack of one SS affects the secretion by other SSs; epithelial cells diminish the effects caused by mutating some SS; bioinformatics analyses identified new potential substrates for each SS. Western blot analysis validated the interdependence in substrate secretion between SSs. Consequently, a complex interaction exists between SSs during epithelial cell infection, affecting the epithelial cells–bacteria interaction and the EHEC pathogenesis.

## Introduction

Enterohemorrhagic *Escherichia coli* (EHEC) belongs to the Shiga toxin-producing *E. coli* (STEC) group. The O157:H7 serotype has been implicated as the leading cause of the human disease [1]. EHEC is an extracellular pathogen characterized by the formation of attaching and effacing (A/E) lesions, upon colonization of the gastrointestinal tract [2]. Clinical manifestation caused by EHEC range from asymptomatic to non-bloody diarrhea or self-limited hemorrhagic colitis. However, 5–7% of cases can be complicated by hemolytic uremic syndrome, and some of these cases can be fatal. EHEC infections are a serious public health problem due to the continued increase in outbreaks worldwide [3–5]. The alarming ability of EHEC to produce infection from a low infectious dose (<100 bacteria) could be associated with the horizontal transfer of virulence factors that allow it to infect efficiently [6,7]. Many virulence factors are encoded in O island (OI), which are genomic islands inserted into the bacterial genome. EHEC harbors 177 OIs comprising 1387 genes, of which only 69 genes (~5%) are associated with virulence, and the function of 1271 genes (~91%) is unknown. Two relevant virulence factors are encoded on these OIs, Shiga toxin (OI-45) and the type three secretion system (T3SS on OI-148), the latter also part of the Locus of Enterocyte Effacement (LEE) [8]. Other key virulence factors are located on the plasmid pO157 of 92 kb. In this plasmid, three secretion systems (SSs) are encoded: T1SS, T2SS, and T5aSS [9]. Furthermore, a pathogenicity island associated with a T6SS is located on the EHEC chromosome [10]. However, only a few EHEC substrates have been reported for all these five SSs.

SSs are nanomachines specialized in the secretion and/or translocation of proteins (substrates), which ensure the delivery of virulence factors during an infectious process. Among the T3SS, 39 effector proteins have been experimentally confirmed, making EHEC the A/E pathogen with the broadest repertoire of proteins associated to this SS [11]. Remarkably, most of these effectors are encoded on lambdoid prophage, highlighting the bacteriophage contribution in the diversity of virulence factors in EHEC. Unlike the T3SS, the other SSs have been underexplored. Despite this, isolated studies have shown that virulence factors associated to cell lysis (HlyA-T1SS), mucin degradation (StcE-T2SS), immune response modulation (StcE-T2SS, EspP-T5aSS, KatN-T6SS), and coagulation factor degradation (EspP-T5aSS) are secreted through these other EHEC SSs [10,12–16]. These reports strongly suggest that SSs play a relevant role during EHEC pathogenesis.

The broad repertoire of proteins secreted by T3SS has been shown to be one of the most important factors for EHEC colonization. However, other SSs could also contribute to EHEC pathogenesis, primarily because their function or activation requires high bacterial energy consumption. Therefore, it is plausible that these macromolecular complexes (T1SS, T2SS) are used for secretion of more than one substrate by each SS. Thereby, it is contradictory that only one substrate for the T1SS has been reported, HlyA [12] or two substrates secreted by the T2SS, StcE, and YodA [13,17]. In fact, proteomics analyses in human pathogenic bacteria have expanded the substrate repertoire secreted by the T2SS [18,19]. Through the T2SS are secreted substrates that fulfill a wide spectrum of functions, such as proteins involved in biofilm formation, immune response modulators, mucinases, lipases, adhesins, and toxins, among others [13,18–21]. Furthermore, in the surrogate model of EHEC, *Citrobacter rodentium* (CR), the absence of T2SS has recently showed leading to colonization defects [22]. Alternatively, these substrates could cooperate with each other, e.g. the interaction between Tir (T3SS) and intimin (Eae [T5eSS]) during bacterial adhesion to host cells [23]. EspP (T5aSS) cleaves HlyA (T1SS), inhibiting cell lysis, suggesting a possible regulation of EHEC virulence through its secreted virulence factors [24]. Interestingly, several substrates secreted by the five EHEC SSs have been described, but how they collectively participate in the interaction with epithelial cells during the infectious process is unknown. In this work, we optimized protein secretion through different EHEC SSs. We determined the secretion hierarchy for the different SSs by using known substrates as well as the effect of the absence or presence of epithelial cells on the protein secretion profile. Finally, we characterized through an extensive proteomic analysis, the secretome of EHEC and different mutants in the SSs (ΔT1SS, ΔT2SS, ΔT3SS). Therefore, here we describe the complex interplay among the different SSs, including their interdependence, the hierarchy of secretion of each SS, the influence of epithelial cells, the lack of secreted substrates due to mutations in the structural proteins of each SS, and the suppression of this lack in some substrates by the presence of epithelial cells. Finally, we analyzed the differentially secreted substrates between mutant and wild-type strains using bioinformatics prediction to propose new potential substrates for the different EHEC SSs.

## Material and methods

### Bacterial strains

All the strains and plasmids used in this study are enlisted in Table 1. EHEC EDL933, Δ*escN* and Δ*espP* were provided by Dr José Luis Puente. pKD4 and pKD46 were provided by Dr Eric Oswald (Table 1). All strains were routinely grown in Luria-Bertani (LB) medium, and when necessary, LB was supplemented with ampicillin (Amp,100 µg/mL), kanamycin (Km, 50 µg/mL) or chloramphenicol (Cm, 25 µg/mL). For experimental assays, the bacteria were grown in DMEM (Dulbecco’s Modified Eagle’s Medium) or our modified M9 (M9 supplemented with 10 mM NaHCO_3_, 5.5 mM D-glucose, 1 mM sodium pyruvate).

**Table 1.** Strains and plasmids used in this study.

Strain or Plasmid	Genotype or Description	Reference
**Strains EHEC**		
O157:H7	Prototype EHEC EDL933 (Wild-type)	[25]
ΔT1SS	EDL933 *ΔhlyD::km*	This study
ΔT2SS	EDL933 *ΔetpC::km*	This study
ΔT3SS	EDL933 *ΔescN::km*	[25]
ΔT5SS	EDL933 *ΔespP::km*	[25]
**Strains *E. coli***		
BL21 (DE3) *pLys*	*E. coli* strain used for recombinant protein expression	Invitrogen
BL21 (p*hlyA*)	Minimal clone *hlyA*	This study
BL21 (p*stcE*)	Minimal clone *stcE*	This study
BL21 (p*espB*)	Minimal clone *espB*	This study
BL21 (p*espP*-PI)	Minimal clone *espP*-PI	This study
**Plasmids**		
pKD46	Plasmid containing Red recombinase system	[26]
pkD4	Plasmid containing the Km cassette for lambda Red recombination	[26]
pTrcHis2B	Inducible vector for recombinant protein expression	ThermoFisher Scientific
pRSETA	Inducible vector for recombinant protein expression	ThermoFisher Scientific
p*hlyA*	*hlyA*, cloned in pTrcHis2B	This study
p*stcE*	*stcE*, cloned in pTrcHis2B	This study
p*espB*	*espB*, cloned in pTrcHis2B	This study
p*espP*-PI	Fragment of nucleotide 169–2370 of *espP*, cloned in pRSET-A	This study

### Mutant construction

To obtain mutants in the SSs, structural genes, such as *hlyD* (T1SS) *etpC* (T2SS), *escN* (T3SS), or *espP* (T5SS), were replaced by a kanamycin resistance cassette according to the homologous recombination method [26]. Briefly, the kanamycin resistance cassette was amplified from plasmid pKD4 using the primers indicated in Table 2. PCR products obtained were used to transform the electrocompetent EHEC, containing the plasmid pKD46 for the expression of the Red λ recombinase. Bacteria growing in LB-Km were selected and, to corroborate the deletion, internal fragments of interest genes were amplified using the primers indicated in Table 2. Finally, plasmid pKD46 was eliminated by growing the mutant strains at 46°C.

**Table 2.** Primers used in this study.

Primer	Sequence (5´-3´)
**Isogenic Mutants**	
hlyD-FRT-F	CAG TTT ATA TGC ATA TTT ATA TCA GTT GCA GGC ATA ACG TTG TAG GCT GGA GCT GCT TCG
hlyD-FRT-R	GCC AAT ATG TTA TTT ATA TAC TAA CGT TCA CGT AAA CTT TCC ATA TGA ATA TCC TCC TTA G
etpC-FRT-F	CCG TTA ATC AAA ACA TAA AGT AGC GTC CAG CGT TTT ATT GTG TAG GCT GGA GCT GCT TC
etpC-FRT-R	TAC CGG CAA ACG CCC ATT CAA CCA TTT CCT GAA CAA TCA CAT ATG AAT ATC CTC CTT AG
**Internal Sequences of Mutated Gene:**	
F-hlyD	TGA CGG TGC CTG GTG TTG AAT CTG
R-hlyD	CCT GAA CAG TAC CAC TCA CAG GAG C
F-etpC	ATC AGC TGG TGT CGG TTA TC
R-etpC	TAC CCG GAA TCA AGC GAT AA
F-escN	TTG CGG TAT TGG GCA GCG TA
R-escN	GTA ACG AAG AAA AAA CAC TCG GAG G
F-espP	CTG TTG GTC GTA ATG CAA AT
R-espP	GAG GTC GCT TTC ATC ATG AC
**Cloning of Recombinant Proteins:**	
F-XhoI-hlyA	CCG CTC GAG CAT GAC AGT AAA TAA AAT A
R-XbaI-hlyA	TGC TCT AGA GTG ACA GTT GTC GTT AAA G
F-XhoI-stcE	CCG CTC GAG CAT GAA CAC TAA AAT GAA TGA
R-KpnI-stcE	CGG GGT ACC GTT TAT ATA CAA CCC TCA TTG
F-XhoI-espB	CCG CTC GAG CAT GAA TAC TAT TGA TAA TAC TCA
R-XbaI-espB	CTA GTC TAG AGT CCC AGC TAA GCG ACC C
F-BamHI-espP-PI	CCG CTC GAG TCA GTT GAC CTC GTT CAG AAA CG
R-NcoI-espP-PI	CAT GCC ATG GTC AGT CGT CAG TCA GTA GAT AAG

### Cloning of recombinant proteins

Plasmid pO157, pTrcHis2B, and pRSET A were purified using the DNA Wizard Plus SV Minipreps. The genes *hlyA, stcE* and *espP* (EspP-PI, see Table 1) were amplified from plasmid pO157, while the *espB* gene was amplified from lysates of EHEC EDL933. PCR products were purified from agarose gels using the DNA QIAquick extraction kit (Qiagen, Inc.). Digestion of PCR products or plasmids (pTrcHis2B or pRSET A) was performed with restriction enzymes indicated in Table 2. After ligation, each construct was transformed into competent *E. coli* TOP10 and selected in LB-Amp. Each construction was confirmed by PCR amplifying each gene.

### Expression and purification of proteins

A colony of *E. coli* BL21 (pTrcHis2B:*hlyA*, pTrcHis2B:*stcE*, pTrcHis2B:*espB*) or *E. coli* BL21 (pRSETA:*espP*-PI) was grown in LB-Amp/Cm broth at 37°C overnight (ON). ON cultures were inoculated into LB-Amp broth at an optical density of 0.1 at 600 nm (OD_600_). Cultures were grown at 37°C with vigorous shaking until an OD_600_ of 0.7–0.9. They were then induced with 1 mM IPTG (isopropyl-β-D-thiogalactopyranoside) for 4 h. The cultures were centrifuged at 4000 × *g* at 4°C for 20 min.

Recombinant proteins were purified using denaturing conditions according to QIAexpress® Ni-NTA (QIAGEN, Inc). Briefly, the *E. coli* cell pellet from a 100 mL culture was sonicated in 10 mL of lysis buffer (0.1 M NaH_2_PO_4_, 10 mM NH_2_C(CH_2_OH)_3_, pH 8.0) for 1 min and then centrifuged at 9000 × *g* at 4°C for 10 min. Pellet was resuspended in a denaturing lysis buffer (0.1 M NaH_2_PO_4_, 10 mM NH_2_C(CH_2_OH)_3_, 8 M NH_2_CONH_2_, pH 8.0), incubated with gentle shaking for 30 min, and subsequently centrifuged at 14,000 × *g* at 4°C for 10 min. The clarified solution was purified on Ni-NTA column; flow-through fraction (unbound) was obtained with wash buffer (0.1 M NaH_2_PO_4_, 10 mM NH_2_C(CH_2_OH)_3,_ 8 M NH_2_CONH_2_, pH 7.5). Finally, 6x His-tagged proteins were eluted with elution buffer (0.1 M NaH_2_PO_4_, 10 mM NH_2_C(CH_2_OH)_3,_ 8 M NH_2_CONH_2_, pH 5–4.5).

### Preparation of polyclonal antibodies

Antibodies against HlyA-T1SS, StcE-T2SS, EspB-T3SS, and EspP-T5aSS were obtained after immunizing BALB/cJ mice with 4.5 µg of purified protein. The protein was injected intramuscularly using TiterMax® as the adjuvant. After the first immunization, three boosters were performed at two weeks intervals. Whole blood was collected by cardiac puncture, and the serum was used in Western blot analysis.

### Protein secretion assays

Wild-type (WT) EHEC and/or isogenic mutant strains were grown ON in LB broth at 37°C under static conditions, and their bacterial growth was compared on a growth curve to ensure the same number of bacteria during secretion assays.

*Activation of different SSs*. Traditionally, for the expression of T3SS, bacterial cultures are activated using DMEM. Thereby, we used different media to activate the different SSs. ON culture of WT-EHEC was subcultured at a 1:20 dilution in DMEM high glucose (DMEM_HG_), DMEM low glucose (DMEM_LG_) or modified M9 (MM9) at 37°C, and 5% CO_2_ for 4 h. Secreted proteins were analyzed as previously described [27], under different culture media for protein secretion detection. Briefly, activated WT-EHEC cultures were inoculated into 250-mL flasks containing 25 mL of DMEM_HG_ or a mixture DMEM_HG_:MM9 (1:1,1:2,1:3), DMEM_LG_ or a mixture DMEM_LG_:MM9 (1:1) or MM9. All cultures were adjusted to OD_600_ of 0.05 and then incubated at 37°C and 5% CO_2_ for 4 h under static conditions. Supernatants were centrifuged at 7000 × *g* at 4°C for 15 min. To remove residuals bacteria, supernatants were filtered through 0.22 µm filters (Sartorius, Göttingen, Germany). Secreted proteins were precipitated ON with 15% trichloroacetic acid and then were separated by SDS-PAGE using Precision Plus Protein^TM^ BIO-RAD (#161–0373) as molecular weight marker. Substrates of different secretion system were analyzed by Western blot using mouse anti-HlyA, anti-StcE, anti-EspP, anti-EspB (this work) or rabbit anti-Tir, anti-EspA (kindly donated by Roxane Piazza) as primary antibodies, and HRP-conjugated anti-isotype as secondary antibody (#G21040, #A16104 Invitrogen). Protein secretion was normalized according to the final optical density of each culture at the indicated secretion times. Bovine serum albumin (BSA) was used as a loading control.

*Protein secretion by WT-EHEC, ΔT1SS, ΔT2SS, ΔT3SS, ΔT5aSS*. All strains were grown ON and each one subcultured at a 1:20 dilution in MM9 at 37°C and 5% CO_2_, for 4 h. Activated cultures were centrifuged at 3000 × *g* for 10 min and each pellet was resuspended with DMEM_HG_ without supplements (medium for detecting the secreted proteins). To compare secreted proteins in the absence or presence of cells, bacteria were inoculated in 60-mm Petri dishes with a final volume of 5 mL and the OD_600_ was adjusted based on the MOI 10 used during infection with cells. Secreted proteins were analyzed as mentioned above.

### Cell culture and infection model

Human epithelial HT-29 cells (ATCC HTB-38) were cultured using DMEM supplemented with 10% fetal bovine serum (HyClone, Logan, UT), 1% nonessential amino acids, 5 mM L-glutamine, penicillin (100 U/mL), and streptomycin (100 µg/mL). Cells were harvested with 0.25% trypsin and 0.53 mM EDTA (Gibco-BRL, Grand Island, NY) in phosphate-buffered saline (PBS [pH 7.4]) and incubated at 37°C in a humidified atmosphere at 5% CO_2_. Confluent HT-29 cells were infected with WT-EHEC or isogenic mutants (multiplicity of infection [MOI] 10) at the indicated times.

### Secretome analysis by LC-MS/MS

Secretome analysis was carried out using supernatant samples from WT-EHEC, ΔT1SS, ΔT2SS, or ΔT3SS strains. Supernatants in the absence or presence of cells were obtained as mentioned above. Secreted proteins were concentrated 80-fold using an Amicon® Ultra-15 filter with a 10 kDa cutoff (Merck, Darmstadt, DEU); thereby we are excluding small proteins lower than 10 kDa in this study. Proteins were prepared for LC-MS/MS using 50 µg of each sample. Proteins were precipitated with methanol-chloroform and then digested as previously described and according to iST® Sample Preparation Kit (PreOmics, Munich, Germany) [28]. Briefly, tryptic digestions were performed for 2 h and subsequently evaporated to dryness in a SpeedVac (ThermoFisher Scientific, Waltham, USA). Samples were resuspended with LC-Load and then normalized to 1 µg/µL. *S. cerevisiae* alcohol dehydrogenase 1 (ADH) was added to a final concentration of 25 fmol/µL.

The analytic method was applied as previously described [29]. Briefly, tryptic peptides were separated on a 75 µm × 150 mm HSS T3 C18 column (100 Å pore size, 1.8 µm particle size (Waters, Milford, MA)). Peptides were eluted on an ACQUITY M-Class UPLC using mobile phase A (0.1% formic acid [FA], in water) and mobile phase B (0.1% FA in acetonitrile [ACN]) using the following gradient: 0 min 7% B, 121.49 min 40% B, 123.15 to 126.46 min 85% B, 129–130 min 7% B, at a flow rate of 400 nL·min^−1^ at 45°C. Spectra data were acquired on a Synapt G2-S*i* (Waters, Milford, MA) using nanoelectrospray ionization (nanoESI) and ion mobility separation (IM-MS) using full-scan DIA approach via High-Definition Multiplexed MS/MS (HDMS^E^) mode. Two chromatograms (low- and high-energy chromatograms) were acquired in positive mode in a m/z range of 50−2000 with a scan time of 500 ms. No collision energy was applied to obtain the low-energy chromatograms, while for the high-energy chromatograms, precursor ions were fragmented in the transfer cell using a collision energy ramp (CID) from 19 to 55 eV. The proteomic analysis included an exploratory experiment and two additional biological replicates, from which we selected a representative biological experiment with the best three technical replicates.

### Mass spectrometry data analysis

The MS and MS/MS measurements contained in the *.raw files were absolutely quantified by Progenesis QI for Proteomics software v4.2 (Nonlinear Dynamics, Newcastle, UK) against a EHEC EDL933 database (*.fasta, obtained from UNIPROT, UP000002519 plus ADH1 from *S. cervisiae*, P00330) [30]. The parameters used for protein identification were trypsin as the cleavage enzyme and allowing up to one missed cleavage. Fixed modification: carbamidomethyl (C) and variable modifications: oxidation (M), amidation (C-terminal), deamidation (Q, N), and phosphorylation (S, T, Y). Acceptance criteria for protein identification were established with a predetermined tolerance for peptides and fragments (maximum normal distribution of 10 and 20 ppm, respectively) and an FDR ≤ 1%. All false positives, as well as proteins with only one identified peptide, were discarded for subsequent analysis. Synapt G2-S*i* was calibrated with [Glu1]-Fibrinopeptide fragments, by fragmentation of the precursor ion [M + 2H]^2+^ = 785.84261 of 32 eV to less than 1 ppm across all MS/MS measurements. Protein quantification was calculated using the Top3 value, which corresponds to the arithmetic mean of the MS signal of the three most intense tryptic peptides for each protein. The amount of protein in the samples was calculated according to the equations previously reported [31,32]. All proteins with ratio ±2 (log2) and *Q-value ≤*0.01 (−log10) were considered differentially secreted.

### Bioinformatic analysis

Proteins identified by LC-MS/MS were analyzed using PSORTb v3.0.3 [33] to determine their subcellular localization. SignalP v6.0 [34] was used to identify the signal peptide (SP) for periplasmic export (Sec or Tat), and SecretomeP v2.0 [35] was used to identify non-classically secreted proteins (NCS). The use of both software programs allows us to infer the type of secretion used by the analyzed protein. The BastionX module of BastionHub software [36] was used to predict new putative substrates for SSs. To analyze secreted proteins, the proteome of EHEC EDL933 was obtained from UNIPROT (UP000002519) in January 2023. Venn diagrams were constructed using the VennDiagram package for R [37].

### Statistical analysis

Statistical analysis was performed using GraphPad Prism v10.0 software (San Diego, USA). Results were expressed as mean ± standard deviation at least three independent experiments. Statistical significance was determined using Student’s *t*-test or ANOVA, and a *p* < 0.05 was considered significant.

## Results

### EHEC secretes proteins according to the activation and secretion media

**Figure 1.**
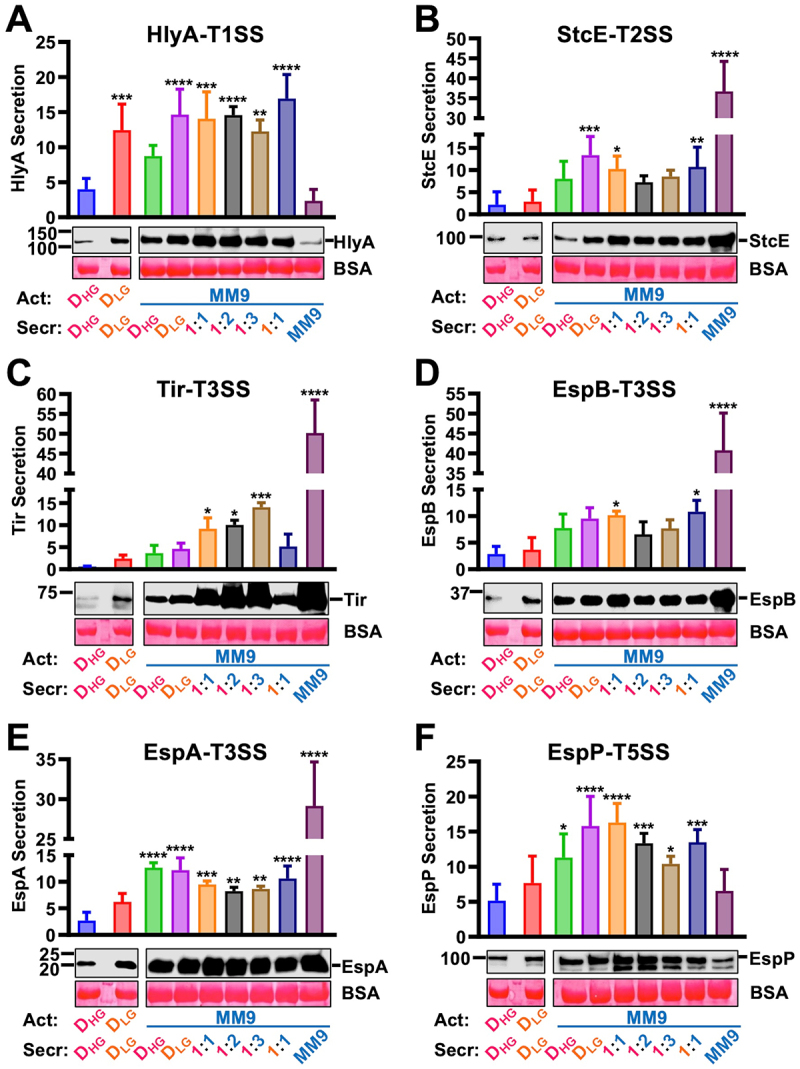
Wild-type EHEC secretes virulence factors by its different secretion systems. EHEC secretes proteins according to the activation and secretion media by T1SS (A), T2SS (B), T3SS (C–E) and T5SS (F). During activation (act), an overnight (ON) culture of WT-EHEC was diluted 1:20 into DMEM high glucose (D_HG_), DMEM low glucose (D_LG_), or modified M9 (MM9) for 4 h at 37°C and 5% CO_2_. for the secretion (secr) analysis, the activated cultures were inoculated in D_HG_, D_LG_, or mixtures of D_HG_:MM9 (1:1, 1:2, 1:3 ratios), D_LG_:MM9 (1:1 ratio), or MM9 alone at OD_600_ = 0.05 for 4 h at 37°C and 5% CO_2_. Supernatants were filtered and the secreted proteins were precipitated with TCA at a final concentration of 15%. The equivalent of 3 mL of supernatant was analyzed by Western blot using antibodies against substrates secreted by T1SS (HlyA), T2SS (StcE), T3SS (Tir, EspA, and EspB), and T5aSS (EspP). Bovine serum albumin (BSA) was added to the supernatants and used as a loading control. Protein secretion levels were normalized to the final OD_600_ of each culture. Data were plotted and are shown as the mean ± SD from four independent experiments. Statistical analyses were performed using one-way ANOVA followed by Dunnett’s test, each treatment was compared against WT-EHEC activated in D_HG_ medium and secreting into D_HG_ (*, *p* < 0.05; **, *p* < 0.01; ***, *p* < 0.001; ****, *p* < 0.0001).

To find an appropriate medium to increase the secretion of most of the virulence factors by EHEC, we used different media formulations to activate EHEC and secrete substrates by the different SSs. Antibodies against specific substrates for each SS were used (T1SS: HlyA, T2SS: StcE, T3SS: Tir, EspA and EspB, and T5aSS: EspP). The formulation media for activating EHEC were the following: DMEM high glucose, DMEM low glucose, or modified M9 (MM9) for 4 h at 37°C and 5% CO_2_. For secretion, the activated cultures were inoculated in DMEM high glucose, DMEM low glucose, or a mixture of DMEM high glucose:MM9 (1:1, 1:2, 1:3 ratios), DMEM low glucose:MM9 (1:1 ratio), or MM9 alone for 4 h at 37°C and 5% CO_2_. The formulation of the secretion medium was necessary to define a medium that had a worthy substrate secretion, but that also allowed the growth of epithelial cells that we used in subsequent experiments. None of these media allowed the secretion of effectors by the T6SS (data not shown). Secretions by T1SS, T3SS, and T5aSS appears to be higher in DMEM low glucose than in DMEM high glucose (Figure 1(A, C–F)) but the increases only reach statistical significance in the case of the T1SS (*p* < 0.001 ANOVA), while Tir and EspA (T3SS) secretions was higher and statistically significant when comparing these two media (*p* = 0.018 and *p* = 0.007, respectively, Student’s *t*-test) (Figure 1). Moreover, the highest secretion of substrates occurred in MM9 medium through the T2SS (StcE) and T3SS (Tir, EspA, and EspB) (Figure 1(B, C–E)) but not through the T1SS (HlyA) and T5aSS (EspP) Figure 1(A,F)). We aimed to understand the secretion of substrates by the SSs during the infection of epithelial cells; however, neither DMEM low glucose medium nor MM9 medium allowed the efficient growth of epithelial cells. Therefore, we used a combination of DMEM high glucose plus MM9 at different ratios after the activation using MM9. An increase in protein secretion was detected by all the SSs when using mixtures 1:1, 1:2, and 1:3 of DMEM high glucose and MM9. Unfortunately, none of these mixtures allowed an optimal growth of the epithelial cells (data not shown). However, all SSs optimally improved when EHEC was activated in MM9 (4 h) plus any eukaryotic cell culture media (i.e. DMEM high glucose) used during the secretion assay. These conditions were used for the secretion throughout subsequent experiments. Moreover, our prepared antibodies specifically recognized six emblematic proteins secreted by the four SSs detectable in the supernatants of EHEC (Figure 1).

### EHEC increases protein secretion by T1SS, T2SS, T3SS, and T5SS during contact with epithelial cells

**Figure 2.**
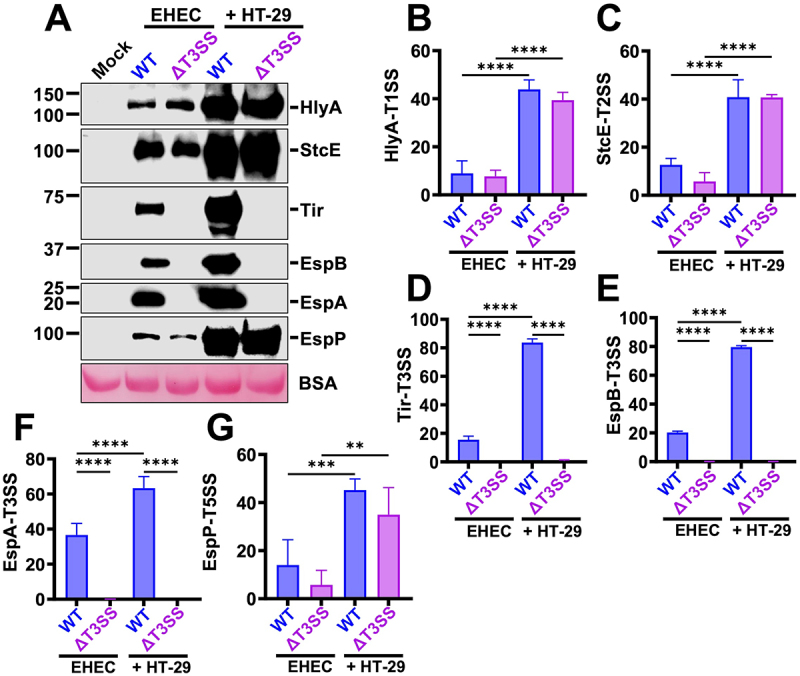
EHEC increases virulence factors secretion during interaction with epithelial cells. (A) Analysis of substrates secreted by the different EHEC secretion systems in either the absence or presence of HT-29 cells. WT-EHEC and ΔT3SS were activated in MM9 during 4 h at 37°C and 5% CO_2_. Activated cultures were inoculated into Petri dishes with D_HG_ adjusted at a MOI of 10 and incubated for 3 h at 37°C and 5% CO_2_. Supernatants were filtered and the secreted proteins were precipitated with 15% TCA. An equivalent of 3 mL of supernatant was analyzed by Western blot using antibodies against substrates secreted by the T1SS (HlyA), T2SS (StcE), T3SS (EspB, EspA, and Tir), and T5aSS (EspP); BSA was added as a loading control. (B-G) Densitometric analyses of the different secreted proteins were normalized with the final OD_600_ of each culture. The plotted data show the mean ± SD from four independent experiments. Statistical analyses were performed using one-way ANOVA followed by Sidak’s correction test (**, *p* < 0.01; ***, *p* < 0.001; ****, *p* < 0.0001).

To characterize the different SSs during the infection, the epithelial cells were infected with WT-EHEC or ΔT3SS (activated in MM9) at MOI 10 for 3 h in DMEM high glucose. For comparison, a similar treatment was used in Petri dishes without epithelial cells. Supernatants were analyzed by Western blot using antibodies against specific substrates of each SSs. In the presence of epithelial cells, EHEC significantly increased protein secretion by all the SSs tested, compared to cultures in the absence of them (Figure 2(A)): approximately four-fold for HlyA (T1SS) (Figure 2(B)), StcE (T2SS) (Figure 2(C)), and EspP (T5aSS) (Figure 2(G)), six-fold for Tir and EspB, and two-fold for EspA (Figure 2(D-F)). As expected, in the ΔT3SS, the tested T3SS effectors (Tir, EspA, and EspB) were not detected in supernatants in the presence or absence of epithelial cells. However, in the presence of epithelial cells, the substrate secretion by T1SS, T2SS, and T5aSS in the ΔT3SS increased as in the WT-EHEC (Figure 2). These data indicate that the SSs are potentiated during epithelial cell infection, and at least the T3SS mutation does not affect the secretion of specific substrates by T1SS, T2SS, or T5aSS.

### Secretion hierarchy through the different EHEC secretion systems

**Figure 3.**
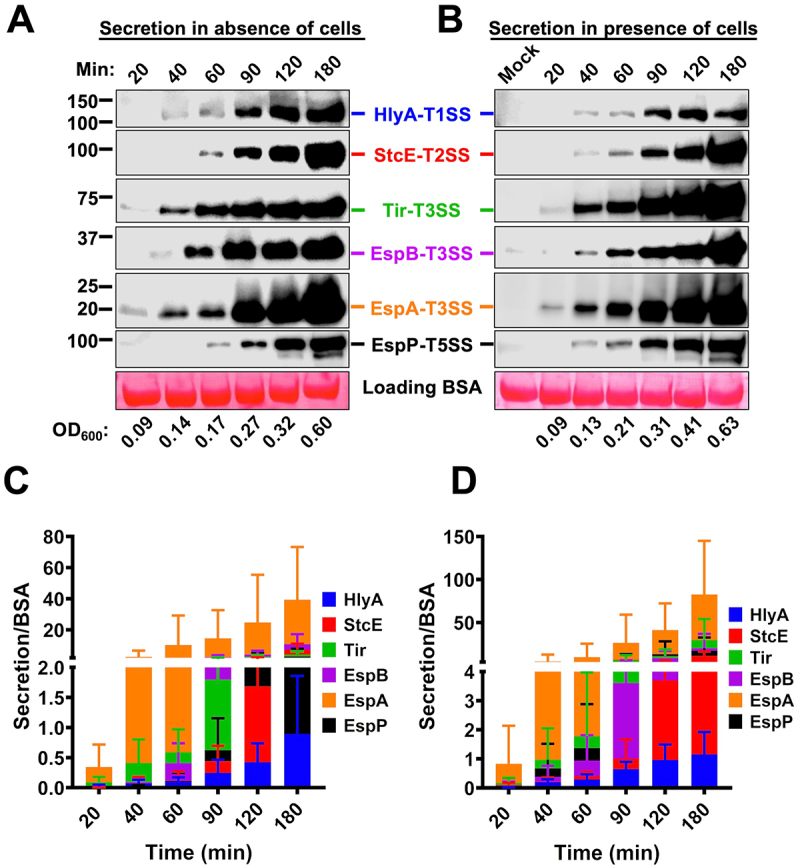
Secretion kinetics through the EHEC secretion systems (SS). (A-B) EHEC differentially secretes virulence factors through T1SS, T2SS, T3SS and T5SS. WT-EHEC was activated in MM9 and then inoculated into Petri dishes with D_HG_ either in the absence or presence of HT-29 cells at a MOI of 10 for 3 h at 37°C and 5% CO_2_. Supernatants were filtered and precipitated with 15% of TCA. An equivalent to 3 mL of supernatant was analyzed by Western blot using specific antibodies against substrates in each SS, BSA was used as a loading control. (C-D) EHEC activates its SS at different times. Band intensities were normalized using BSA signal. The data were plotted, and each point represents the mean ± SD from four independent experiments. The number presented at the bottom of the panel A and B represent the bacterial growth detected by OD_600_ reached at the indicated times.

To investigate which SSs are the first to be activated during EHEC-epithelial cell interaction, and the secretion hierarchy by the different SSs, we performed secretion kinetics. HT-29 cells were infected at various times from 20 to 180 min, and the supernatants were analyzed by Western blot using antibodies against HlyA, StcE, Tir, EspA, EspB, or EspP. For comparison, similar experiments were performed without HT-29 cells (EHEC in the absence of cells). The T3SS (mainly through EspA and Tir secretion) was the first SS to be activated in the presence of epithelial cells as well as in their absence (20 min) (Figure 3(A,C)). The second system activated was the T1SS (40 min), while the T2SS and T5aSS were the last to be activated (60 min). During the infection of epithelial cells, all the SSs were activated at 40 min of infection, except for the T3SS that was active since 20 min of infection (Figure 3(B,D)). Thus, the substrate secretion by T2SS and T5aSS was improved by 20 min in the presence of epithelial cells. In all cases, in the absence (Figure 3(C)) or presence of epithelial cells (Figure 3(D)), the increase in concentration of substrates in supernatants was time-dependent, the highest increase was in the presence of cells (Figure 3(D) vs 3C), and the highest concentration of proteins was detected for the substrates from T3SS and T2SS.

### Proteins secreted by EHEC and mutants in T1SS, T2SS, and T3SS

**Figure 4.**
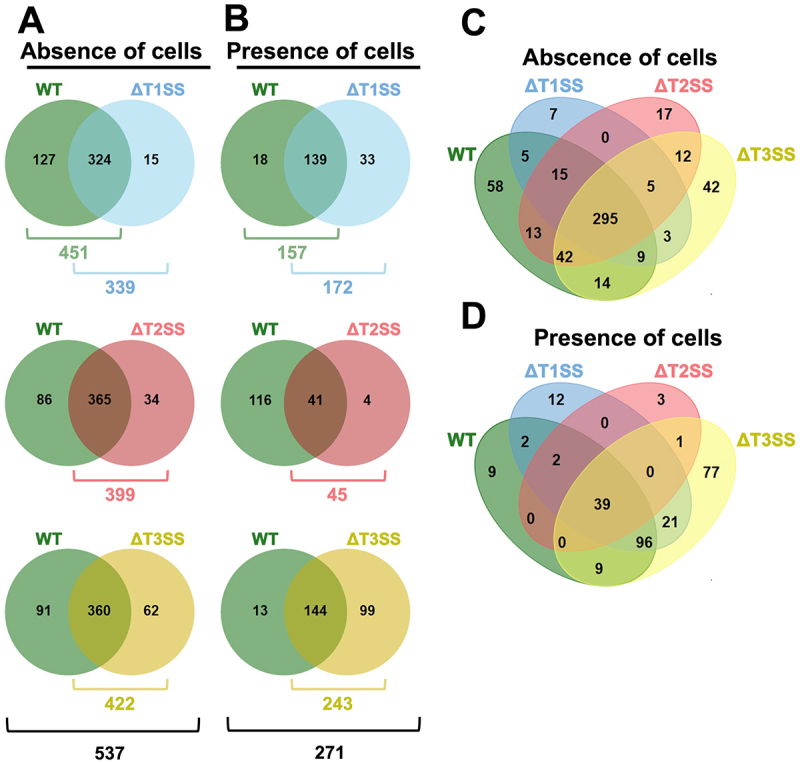
Venn diagrams showing the comparison between proteins from the supernatant of WT-EHEC and its mutants in specific secretion systems. (A-B) supernatants analysis by LC-MS/MS of WT-EHEC, ΔT1SS, ΔT2SS, or ΔT3SS either in absence or presence of HT-29 cells. The raw MS/MS data of each supernatant were analyzed and compared using ProteinLynx global SERVER (PLGS) software against EHEC EDL933 database (UniProt). All protein identification reported here showed 50–95% reliability (yellow auto-curate) or ≥95% reliability (green auto-curate) and were obtained from three technical replicates from one representative biological experiment (see M&M section). Venn diagrams represent overlap between WT-EHEC vs each mutant (ΔT1SS or ΔT2SS or ΔT3SS) in the absence (A) or presence of cells (B). (C-D) Venn diagrams showing the overlap of proteins from supernatants identified in all the strains analyzed in the absence (C) or presence of cells (D).

Since the interaction of EHEC-epithelial cells altered the secretion profile of specific substrates by three types of SSs, we decided to analyze the secretome of WT-EHEC, ΔT1SS, ΔT2SS, and ΔT3SS in the absence or presence of epithelial cells. Mutants in T5SS and T6SS were excluded because the proteins secreted by T5SS (autotransporter) are secreted individually since they do not pass through a collective secretion machinery, and T6SS is not expressed *in vitro* by EHEC. Supernatants of strains used (3 h of secretion) analyzed by LC-MS/MS were compared using ProteinLynx Global Server (PLGS) against the EHEC EDL933 database. WT-EHEC changed its secretion profile when infecting epithelial cells. In the absence of cells, WT-EHEC secreted 451 identified proteins, while in the presence of cells only 157 proteins were secreted (Figure 4(A,B)). This decrease in the secreted proteins was also observed for ΔT1SS (339 vs 172), ΔT2SS (399 vs 45), and ΔT3SS (422 vs 243); it was more evident in ΔT2SS. The total proteins identified under all conditions in the absence or presence of cells were 537 and 271, respectively.

By comparing WT-EHEC with each of the mutants, in the absence of cells ΔT1SS shared 324 proteins and 15 were unique for this mutant and 127 for WT-EHEC (Figure 4(A)). In the presence of cells, ΔT1SS shared 139 proteins and 33 were unique for this mutant and 18 for WT-EHEC (Figure 4(B)). In the case of ΔT2SS, in the absence of cells, it shared 365 proteins with WT-EHEC and 34 were unique for this mutant and 86 for WT-EHEC (Figure 4(A)). In the presence of cells, ΔT2SS shared 41 proteins and only four were unique for this mutant and 116 for WT-EHEC (Figure 4(B)). For ΔT3SS, in absence of cells, it shared 360 proteins with WT-EHEC and 62 were unique for this mutant and 91 for WT-EHEC (Figure 4(A)). In the presence of cells, ΔT3SS shared 144 proteins and 99 were unique for this mutant and 13 for WT-EHEC (Figure 4(B). Interestingly, in the absence of cells ΔT2SS shared a larger number of secreted proteins with WT-EHEC (365 from 399, representing 91%). ΔT1SS shared 324 proteins with WT-EHEC, from 339 secreted by this mutant (95%), while ΔT3SS shared 360 proteins from 422 secreted by this mutant (85%) (Figure 4(A,B)). These data strongly suggest that a mutation in T3SS affects a larger number of secreted proteins, whereas a mutation in T1SS has a smaller effect on the number of secreted proteins. In the presence of cells, these ratios change; ΔT3SS shared a smaller number of secreted proteins with WT-EHEC (144 out of 243, 59%), ΔT1SS shared 139 from 172 proteins with WT-EHEC (80%), whereas ΔT2SS shared 41 from 45 (91%). Therefore, ΔT3SS again affected the largest number of secreted proteins, but now ΔT2SS has a smaller effect on the number of secreted proteins than ΔT1SS.

The analyses used to compare the WT-EHEC with the three mutants showed that in the absence of cells, only 58 secreted proteins were not found in SS mutants and this number was reduced to nine proteins in the presence of cells (Figure 4(C,D)). At the same time, 295 secreted proteins were constantly secreted by all (WT-EHEC and mutants) and were reduced to 39 proteins in the presence of cells. From 15 proteins secreted differentially by ΔT1SS in the absence of cells compared with WT-EHEC, five were shared with ΔT2SS and ΔT3SS, and three with ΔT3SS. Whereas in the presence of cells, from the 33 proteins secreted differentially by ΔT1SS vs WT-EHEC, 21 were shared with ΔT3SS. From the 34 proteins secreted differentially by ΔT2SS vs WT-EHEC, five proteins were shared with ΔT1SS and ΔT3SS, and 12 with ΔT3SS. Whereas in the presence of cells, of the four proteins secreted differentially by ΔT2SS vs WT-EHEC, one was shared with ΔT3SS. Finally, from the 62 proteins secreted differentially by ΔT3SS vs WT-EHEC, five proteins were shared with ΔT1SS and ΔT2SS, three proteins with ΔT1SS, and 12 proteins with ΔT2SS. Whereas in the presence of cells, from the 99 proteins secreted differentially by ΔT3SS vs WT-EHEC, one was shared with ΔT2SS and 21 with ΔT1SS (Figure 4(C,D)). These data suggest an intricate network of proteins whose secretion is being affected by the mutation of some SSs.

### Differential protein secretion by WT-EHEC and ΔT1SS

**Figure 5.**
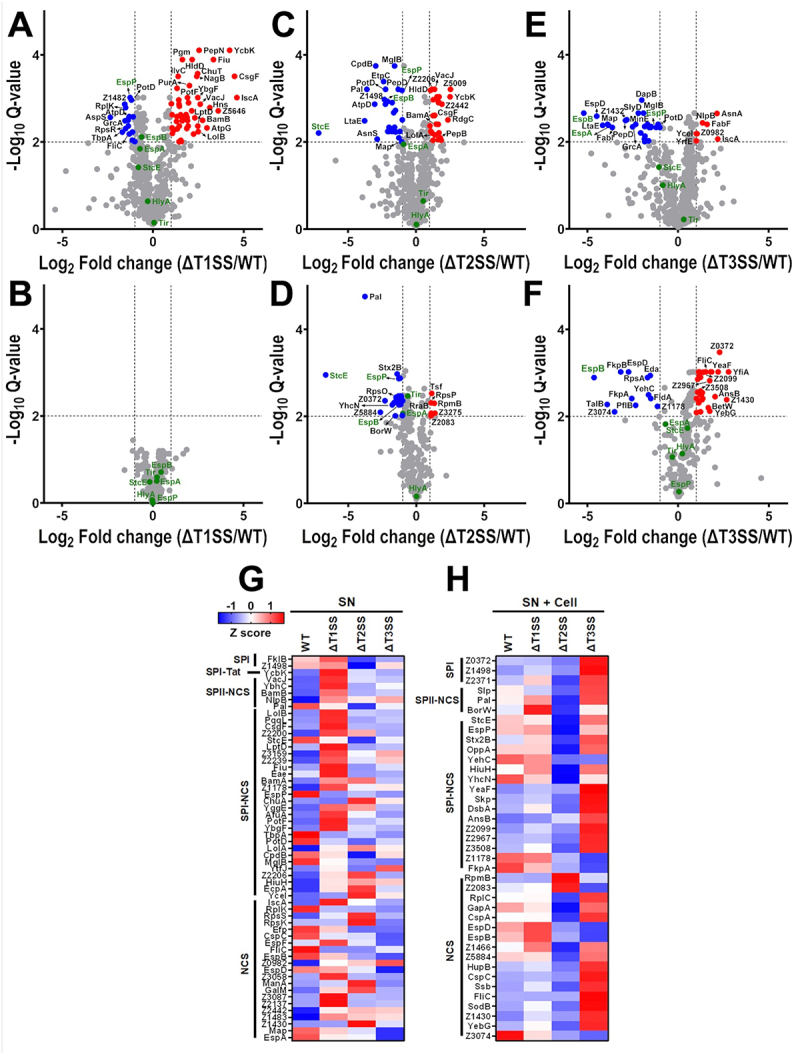
Differential secretion of proteins in absence or presence of cells by WT-EHEC, ΔT1SS, ΔT2SS, or ΔT3SS. raw MS/MS data were quantified using progenesis QI software. Volcano plot of pairwise comparison of the *Q-value* – (log10) vs abundance of proteins log2 between ΔT1SS/WT-EHEC (A), ΔT2SS/WT-EHEC (C), and ΔT3SS/WT-EHEC (E) in the absence or presence of cells (B, D, and F, respectively). Reduced secreted proteins (RSP) are highlighted in blue and increased secreted proteins (ISP) in red, dotted lines represent significance (FC ≥ 1 or ≤−1, FDR < 0.01). Proteins highlighted in green represent hallmark substrates associated to different secretion systems. Heatmaps of z-scored protein abundance of RSP and IPS in the absence of cells (G) or presence of cells (H), categorized by export signal peptide (SP) predicted by SignalP v6.0 and non-classically secreted proteins predicted by SecretomeP v2.0. All analyzed proteins contained at least one unique peptide identified, obtained from three technical replicates from one representative biological experiment (see M&M section). Statistical analyses were performed using Student’s *t-*test followed by Benjamini–Krieger–Yekutieli’s false discovery rate (FDR) of 1%.

To understand which secreted proteins were affected in the absence of T1SS, T2SS, or T3SS, we analyzed all mutants individually in comparison to the WT-EHEC. We also analyzed the kind of signal sequences for protein secretion as an indicator for predicting the SS that the substrate could use. In the case of ΔT1SS in the absence of epithelial cells, numerous proteins displayed altered secretion; 51 proteins were significantly increased and 17 reduced (Figure 5(A)). Among the top 10 most abundant increased proteins (Table S1), and by using SignalP and SecretomeP database to predict signal sequences, four harbor a signal peptide (SP) plus a non-classical secretion prediction (NCS): CsgF, Fiu (SPI and NCS), BamB, and VacJ (SPII and NCS), one with a SP: YcbK (SPI-Tat), and another with an NCS: IscA. Whereas the remaining four were classified as cytoplasmic proteins by PSORTb. Conversely, among the top 10 reduced proteins (Table S1), only one of them harbors SPI and NCS, TbpA, and one harbors an NCS, RplK. The rest of them (eight) were classified as cytoplasmic proteins. Furthermore, from the top 10 increased proteins with the highest statistical difference (–log_10_ Q) (Table S2), HldD, Pgm, ChuT, NagB, IlvC, and PurA were not classified by SignalP and SecretomeP and were classified as cytoplasmic proteins by PSORTb, but ChuT was classified as a periplasmic protein. From the top 10 reduced proteins with the greatest statistical difference (Table S2), EspP harbors SPI and NCS (T5aSS) and was classified as an extracellular protein, PotD (SPI and NCS) and RplK (NCS) were classified as periplasmic and cytoplamic proteins, respectively. SucB was not detected by SignalP or SecretomeP and was classified as a cytoplasmic protein by PSORTb. Clearly, T1SS deletion affects at least one known protein secreted by T5aSS, EspP, and other proteins participating in other SSs: CsgF (curli), BamB (Bam Complex), ChuT (ABC transporter complex), VacJ (its absence is associated with outer membrane vesicle formation; affecting proteins secreted by vesicles) or an important master gene regulator (HNS), whose regulation includes many secreted proteins.

Fascinatingly, protein secretion by ΔT1SS in the presence of epithelial cells completely changed the protein secretion profile. In ΔT1SS in comparison with the WT-EHEC, none of the previously secreted proteins in the absence of cells were significantly reduced or increased (Figure 5(B)), indicating a compensation phenotype by the presence of epithelial cells.

Of all differentially secreted proteins (157) among WT-EHEC, ΔT1SS, ΔT2SS, and ΔT3SS in the absence of epithelial cells, 57 harbored a known export signal: 27 harbored both SPI and NCS, six SPII and NCS, 21 only NCS, two only SPI and one SPI-Tat (Figure 5(G)). From these 57 secreted proteins, 35 (30 increased and five reduced) were differentially secreted in ΔT1SS in comparison with WT-EHEC: 20 harbored both SPI and NCS, 5 SPII and NCS, 9 only NCS, and 1 SPI-Tat (Table S3). Interestingly, for ΔT1SS, only nine proteins harbored NCS (seven increased and only two reduced), while the vast majority seemed to have a periplasmic intermediate (25 SPI/II and NCS). On the other hand, in the presence of cells, from the 72 differentially secreted proteins, 39 harbored a known exported signal: 16 harbored both SPI and NCS, three SPII and NCS, 17 only NCS, and three only SPI (Table S4). From these 39 secreted proteins none was increased or reduced, thereby the presence of cells appears to compensate for the increases and reduction of proteins seen in the absence of cells (Figure 5(H)).

### Differential protein secretion by WT-EHEC and ΔT2SS

By analyzing the proteins secreted by the WT-EHEC in comparison to ΔT2SS in the absence of epithelial cells, 27 proteins were significantly increased and 28 reduced (Figure 5(C)). Among the top 10 most abundant increased proteins (Table S5), one harbored SPI and NCS, YecI, another one only SPI-Tat, YcbK, and two only NCS, Z2442, and RpsK. Whereas the remaining six were classified as cytoplasmic proteins. On the other hand, among the top 10 reduced proteins (Table S5), three of them harbor SPI and NCS (StcE, CpdB, and PotD), one SPII and NCS (Pal), one SPI (Z1498), and another one NCS (Efp). The rest (four) were classified as cytoplasmic proteins.

Additionally, from the top 10 increased proteins with the highest statistical difference (–log_10_ Q) (Table S6), CcdA, HldD, and DeoD were not classified by SignalP or SecretomeP and were classified as cytoplasmic proteins. While VacJ and Z2206 harbored SPII and SPI, and both NCS, and were classified as outer membrane and unknown proteins, respectively. On the other hand, from the top 10 reduced proteins with the greatest statistical difference (Table S6), CpdB, MglB, PotD, and EspP (T5aSS) harbored SPI and NCS, and were classified as periplasmic proteins, except EspP that is extracellular, EspB (NCS), an T3SS effector, was classified as an extracellular protein, and SucC was not detected by SignalP or SecretomeP and was classified as a cytoplasmic protein by PSORTb. Clearly, the T2SS deletion affects at least four well-known substrates secreted by T2SS (StcE), T3SS (EspB), and T5aSS (EspP), including Map (T3SS), which did not reach the top 10 of differentially secreted proteins, as well as other proteins participating in SSs, such as CsgF (curli), BamA (Bam Complex), and Z2206 (adhesin similar to FimH).

Interestingly, and unlike ΔT1SS, ΔT2SS in the presence of epithelial cells differentially secreted 31 proteins as compared to WT-EHEC (Figure 5(D)). However, the presence of epithelial cells did not compensate for the increase or decrease in secreted proteins, which was shown when ΔT2SS was in the absence of cells. Thereby, only six secreted proteins were increased (vs 27 in the absence of cells) and a similar number of secreted proteins was reduced (25 proteins vs 28 in the absence of cells) (Table S7). None of the six proteins identified (Table S7) were previously detected in the absence of cells. Furthermore, the reduced secreted proteins (Table S7) in the presence of epithelial cells were also different proteins except for StcE and Pal. Furthermore, the top 10 increased proteins with the highest statistical difference included only six proteins (Table S8). Tsf, RpsP, RraB, and Z3275 were not classified by SignalP and SecretomeP and were classified as cytoplasmic proteins, except for Z3275, which was classified as an unknown protein. Whereas RpmB and Z2083 harbor an NCS and were classified as cytoplasmic and unknown proteins, respectively. On the other hand, from the top 10 reduced proteins with the greatest statistical difference (Table S8), Stx2B, StcE (T2SS) and EspP (T5aSS) harbored SPI and NCS, Pal harbors SPII and NCS, and GapA, EspD, and RplC only NCS. All of them were classified with specific subcellular localization by PSORTb: as cytoplasmatic (GroEL, GapA, RpsG, RplC, and RpsO), periplasmic (Stx2B), outer membrane (Pal and EspP), or extracellular (StcE and EspD) (Table S8).

In the absence of cells, from the 57 secreted proteins (out of 157) differentially secreted by the three mutants harboring a known export signal, 29 (17 increased and 12 reduced) were differentially secreted in ΔT2SS in comparison with the WT-EHEC: 14 harbor both SPI and NCS, two SPII and NCS, 10 only NCS, two only SPI, and one SPI-Tat (Figure 5(G)). Interestingly, for ΔT2SS, the vast majority (19 proteins) had a periplasmic intermediate: 14 harbor both SPI and NCS (nine increased and five reduced), two SPII and NCS (one increased and one reduced), two SPI (both reduced), or one SPI-Tat (increased), and only 10 proteins harbored NCS (six increased and four reduced) (Table S3). On the other hand, in the presence of cells (Table S4), from the 39 secreted proteins (out of 72) differentially secreted by all the mutants harboring a known export signal, 20 (two increased and 18 reduced) were differentially secreted in ΔT2SS in comparison with the WT-EHEC: seven harbored both SPI and NCS (all of them reduced), three SPII and NCS (all reduced), nine only NCS (two increased and seven reduced), and one only SPI (reduced) (Figure 5(H)).

### Differential protein secretion by WT-EHEC and ΔT3SS

The analysis of proteins secreted by ΔT3SS in comparison to the WT-EHEC in the absence of epithelial cells showed that seven proteins were significantly increased and 27 reduced (Figure 5(E)). However, the number of proteins was smaller than that shown by ΔT1SS and ΔT2SS. Among the top 10 most abundant increased proteins (Table S9), one of them harbor both SPI and NCS, YecI, another only SPII and NCS, NlpB, and two only NCS, IscA and Z0982. Whereas the remaining three were classified as cytoplasmic proteins. On the other hand, among the top 10 most abundant reduced proteins (Table S9), none of them harbor a classical SP, on the contrary four proteins harbor only NCS (EspB, EspD, Map, and EspA). The rest of the proteins (six) were classified as cytoplasmic proteins.

Additionally, from the top 10 increased proteins with the highest statistical difference (Table S10), NlpB harbors both SPII and NCS, YceI SPI and NCS, Z0982 and IscA only NCS, and the other three were classified as cytoplasmic proteins. Whereas, from the top 10 reduced proteins with the greatest statistical difference (Table S10), MglB and PotD harbors SPI and NCS (classified as periplasmic proteins), EspB and EspD (T3SS) harbor NCS (classified as extracellular proteins), and the rest of the proteins (six) were classified as cytoplasmic proteins. Clearly, the T3SS deletion affects the secretion of proteins more than T1SS or T2SS mutation; importantly, it affects the secretion of EspP (T5aSS) and NlpB (Bam complex), this latter could affect indirectly the assembly of the autotransporter barrel. As expected, ΔT3SS also affected several T3SS effectors, in the proteins mentioned above, and beyond the top 10 proteins.

Interestingly, and unlike ΔT1SS, the number of secreted proteins by ΔT3SS in the presence of epithelial cells increased more than in the absence of cells, mainly in those increased proteins, which were 29 proteins as compared to WT-EHEC (vs seven in the absence of cells) (Figure 5(F)). But a smaller number of secreted proteins was reduced (12 proteins vs 27 in the absence of cells). Interestingly, the top 10 increased proteins (Table S11) were differentially secreted proteins (and beyond top 10), since none of the proteins identified were previously detected in the absence of cells. Furthermore, the top 10 reduced secreted proteins (Table S11) in the presence of epithelial cells were also different except for EspB and EspD.

Furthermore, the top 10 increased proteins with the highest statistical difference (Table S12) were classified as harboring both SPI and NCS (YeaF and Z2099), SPI (Z0372), and NCS (CspC and FliC), CspC was classified as a cytoplasmic protein, YeaF, as an outer membrane protein, FliC, as an extracellular protein and Z0372 and Z2099 of unknown subcellular localization. Instead, from the top 10 reduced proteins with the greatest statistical difference (Table S12), YehC and FkpA harbor SPI and NCS, and EspB and EspD an NCS. All of them were classified as cytoplasmatic (FkpB, Eda, RpsA, FldA, TalB, and PflB), as periplasmic (YehC and FkpA), or extracellular (EspD and EspB) proteins (Table S12).

Again, from the 57 secreted proteins (out of 157) differentially secreted by all the mutants harboring a known export signal, unlike the other mutants, in ΔT3SS only 15 (four increased and 11 reduced) were differentially secreted in comparison to the WT-EHEC; five harbored both SPI and NCS, one SPII and NCS and nine only NCS. Interestingly, for ΔT3SS, most proteins harbored an NCS (two increased and seven reduced), and few proteins harbored both SPI (one increased and four reduced) or SPII (one increased) and NCS (Figure 5(G)). On the other hand, in the presence of cells (Table S4), from the 39 secreted proteins (out of 72) differentially secreted by all the mutants harboring a known export signal, 23 (17 increased and six reduced) were differentially secreted in ΔT2SS in comparison to the WT-EHEC: 10 harbored both SPI and NCS (seven increased and three reduced), 10 only NCS (seven increased and three reduced), and three only SPI (all reduced) (Figure 5H).

### Detection of putative novel substrates secreted through T1SS, T2SS, and T3SS

To detect new possible substrates of the different SSs, we analyzed those proteins identified in the supernatants of WT-EHEC by comparing their absence in each SS mutant. Then, they were selected using the following characteristics: to harbor a signal sequence (SignalP and/or SecretomeP), the subcellular localization (PSORTb), not included in our database of known substrates of SSs, not increased in the mutant, and positive in the analyses to predict potential secreted substrate (BastionHub). Thus, we identified 16 potential substrates not previously detected and secreted by EHEC or its mutants: nine were secreted in the absence of cells (five periplasmic proteins and four unknown localizations) and seven secreted in the presence of cells (all of them of unknown localization) (Table 3). Interestingly, one protein, Z2083 (unknown function), which was not identified in the supernatants of both ΔT1SS and ΔT3SS, however, its prediction score by BastionHub was higher for substrate secreted by T3SS (0.968) than for the T1SS (0.665); this result suggests some relationship between the T3SS and T1SS for the secretion of Z2083 (protein Dinl-like 1: DNA damage inducible protein 1). On the other hand, most of the putative novel substrates were secreted by T2SS, detected by BastionHub (13 out of 16: seven in the absence of cells and six in the presence of cells), and the remaining three were predicted to be substrates of T3SS (two in the absence of cells and one in the presence of cells). It is also remarkable that almost 50% of the proteins (seven: four in the absence of cells and three in the presence of cells) are of unknown function (Table 3). From these seven proteins, five are encoded in bacteriophage (Z1452: BP-933W, Z3307: CP-933 V, Z2083: CP933O, Z2967: CP:933T, and Z0955: CP-933K) and four of them were published [8] to be encoded in virulence-related O islands: Z1452 (OI-45, island encoding *stx2A*, *stx2B*), Z3307 (OI-93, island encoding *stx1A* and *stx1B*), Z2083 (OI-57, island encoding *nleG2-3*, *nleG6-2*, and *nleG5-2*), and Z0955 (OI-36, island encoding *nleC*, *nleH1*, and *nleD*).

**Table 3.** List of possible new substrates secreted by EHEC.

ID	Name	SignalP	SecretomeP	PSORTb	Secretion System (SS)	BastionHub Prediction						Absent in
						T1SS	T2SS	T3SS	T4SS	T6SS	Possible SS	
**Proteins identified in absence of cells**												
A0A0H3JEL9	Z2789	SPII	NCS	Periplasmic	Unknown	0.168	0.55*	0.114	0.065	0.103	II	ΔT2SS
A0A6M7H4H9	TbpA	SPI	NCS	Periplasmic	Unknown	0.129	0.568*	0.069	0.056	0.05	II	ΔT2SS
Q8XBT0	Rna	SPI	NCS	Periplasmic	Unknown	0.214	0.992*	0.105	0.133	0.108	II	ΔT2SS
A0A6M7H2J5	UshA	SPI	NCS	Periplasmic	Unknown	0.277	0.853*	0.028	0.046	0.031	II	ΔT2SS
Q8XDX6	Z2786	SPI	NCS	Unknown	Unknown	0.097	0.763*	0.08	0.038	0.035	II	ΔT2SS
Q8X5W6	MalM	SPI	NCS	Periplasmic	Unknown	0.054	0.945*	0.066	0.072	0.054	II	ΔT2SS
Q8X7A0	YnfB	SPI	NCS	Unknown	Unknown	0.324	0.876*	0.346	0.322	0.363	II	ΔT2SS
A0A6M6W104	Z1452	OTHER	NCS	Unknown	Unknown	0.224	0.196	0.588*	0.233	0.391	III	ΔT3SS
Q8XEC0	Z3307	OTHER	NCS	Unknown	Unknown	0.809*	0.704	0.624	0.474	0.884	I II III VI	ΔT3SS
**Proteins identified in presence of cells**												
P58217	Z2083	OTHER	NCS	Unknown	Unknown	0.665	0.411	0.968*	0.923	0.947	I III IV VI	ΔT1SS/ΔT3SS
A0A4P8B947	Z2967	SPI	NCS	Unknown	Unknown	0.345	0.994*	0.127	0.689	0.34	II IV	ΔT2SS
A0A4P8B779	YeaF	SPI	NCS	Outer Membrane	Unknown	0.245	0.745*	0.055	0.067	0.038	II	ΔT2SS
Q8X9X2	YncE	SPI	NCS	Unknown	Unknown	0.116	0.671*	0.067	0.047	0.067	II	ΔT2SS
Q8X881	Z0955	SPI	NCS	Unknown	Unknown	0.411	0.888*	0.093	0.279	0.237	II	ΔT2SS
P58603	OmpT	SPI	NCS	Outer Membrane	Unknown	0.247	0.757*	0.053	0.082	0.108	II	ΔT2SS
A0A6M6W7P0	BorW	SPII	NCS	Unknown	Unknown	0.119	0.719*	0.042	0.21	0.057	II	ΔT2SS

*The highest prediction scores for the secretion systems.

In addition to the proteins mentioned above, which were predominantly classified by BastionHub (plus some extra features), we also searched manually for those proteins that reach several candidate features. Thus, based mainly on the signal sequence, SS unknown, and subcellular localization (no cytoplasmic protein), we found 35 candidate substrates (15 in the absence of cells and 20 in the presence of cells) (Table S13). Most of them (21 proteins) harbored SP and NCS. Therefore, these proteins could be secreted by T2SS but not by T5SS, since the size of these proteins is too small to be secreted by T5SS, or by T4SS, since this SS is not encoded in EHEC. Among the 14 remaining proteins (from 35 candidates), 12 harbored only one NCS, and interestingly, nine were secreted in the presence of cells and only three in the absence of cells. The latter three proteins were classified as unknown localization by PSORTb, while of the nine secreted proteins in the presence of cells, four were classified as extracellular proteins, one as an outer membrane protein and four as unknown localization. Eleven of the 12 proteins (three secreted in the absence of cells and eight in the presence of cells) were of unknown function and the remaining one was SodB, while the other two unknown proteins harbor both SPI and NCS. Thus, from these 14, 13 proteins were unknown, 10 proteins are encoded in bacteriophages: Z3091: CP-933 U, Z3087: CP-933 U, Z1793: CP-933N (in the absence of cells) and Z1382: CP-933 M, Z6027: CP-933P, Z0982: CP-933K, Z2340: CP-933 R, Z3342: CP-933 V, Z1483: BP-933W, Z1466: BP-933W (in the presence of cells). From these 10 proteins, eight were published [8] to be encoded in virulence-related O islands: Z3091 and Z3087 (OI-79, island encoding *espJ* and *espFu*), Z1793 (OI-50, island encoding *nleL* and *espK*), Z6027 (OI-71, island encoding *espM*1, *nleF, nleH2*, and *nleA*), Z0982 (OI-36, island encoding *nleC, nleH1*, and *nleD*), Z3342 (OI-93, island encoding *stx*1A and *stx*1B), Z1483, and Z1466 (OI-45, island encoding *stx*2A and *stx*2B). The remaining two proteins, to complete the 35 candidates, harbor only an SPI, one in the absence of cells (NikA) and the other one in the presence of cells (Stx2A). Interestingly, NikA was predicted to be a T2SS susbtrate by BastionHub despite not having an NCS; additionally, it was not identified in the ΔT2SS supernatant. Whereas Stx2A was not found in the ΔT1SS supernatant and was predicted as secreted by T3SS, T4SS, T6SS, T2SS, and T1SS by BastionHub, in that order. However, it is well known that it is released by cell lysis.

### Dynamic of EHEC, ΔT1SS, ΔT2SS, and ΔT3SS secretion systems

**Figure 6.**
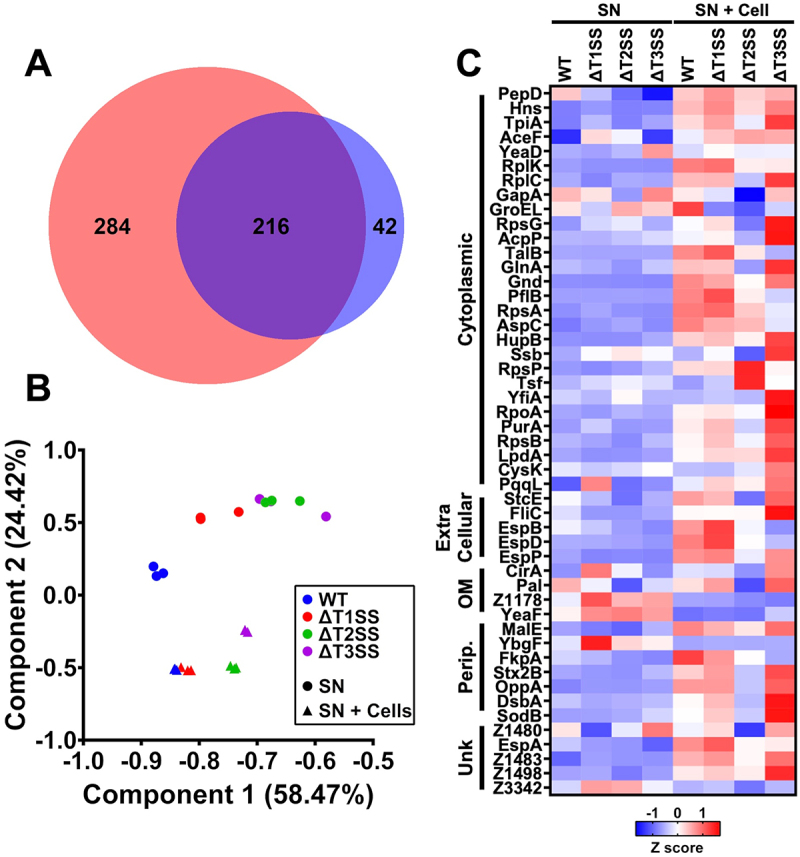
Evaluation of the shared EHEC secretome in the absence or presence of epithelial cells. the proteins from the supernatants were collected in the absence or presence of cells, and the progenesis QI software was used for their quantification and comparison. (A) Venn diagram representing the overlap of identified proteins in EHEC secretome in the absence of cells (red) vs secretome in the presence of cells (blue). (B) Principal component analysis of 216 proteins identified in secretomes of WT-EHEC, ΔT1SS, ΔT2SS or ΔT3SS in the absence (SN) or presence of cells (SN + cells). (C) Heatmaps of z-score differentially secreted proteins between the four secretomes in the absence or presence of cells, categorized by subcellular localization predicted by PSORTb v3.0.3 statistical analyses were performed from three technical replicates from a representative biological experiment using two-way ANOVA followed by Benjamini-Krieger-Yekutieli’s FDR of 1%.

Due to previous findings regarding changes in protein secretion by WT-EHEC and its mutants (by Western blot and proteomics), we performed a principal component analysis (PCA) of the total proteins secreted by WT-EHEC, ΔT1SS, ΔT2SS, and ΔT3SS. The global analysis showed that independently of the mutations, EHEC secreted 542 proteins, 500 proteins were secreted in the absence of cells and 258 were secreted in the presence of epithelial cells, sharing 216 secreted proteins. Thus, 284 proteins were differentially secreted in the absence of cells and only 42 proteins were differentially secreted in the presence of epithelial cells (Figure 6(A)). Components 1 (58.4%) and 2 (24.4%) accounted for 82.8% of the total variance within the data, and all replicates resolved into their corresponding samples. Thus, the PCA clustered WT-EHEC separately from its mutants, with high concordance between replicates (Figure 6(B)). Protein secretion by ΔT2SS and ΔT3SS appeared clustered together while the secretion profile by ΔT1SS appeared slightly separated from the other two mutants, between them and the WT-EHEC. Fascinatingly, the PCA for the secretion of the strains in the presence of cells was different than in the absence of cells; clearly three clusters were identified, the first one clustering the WT-EHEC and ΔT1SS, and the other two clusters belonging to ΔT2SS and ΔT3SS, with very high concordance between replicates.

Of the 216 shared secreted proteins among all strains in the absence and presence of cells, 49 proteins were differentially secreted between WT-EHEC and its mutants (Figure 6(C)). Most of these proteins analyzed by PSORTb were cytoplasmic proteins (28), while the rest were extracellular (five: StcE, FliC, EspB, EspD, and EspP), outer membrane (four: CirA, PaI, Z1178, and YeaF), periplasmic (seven: MalE, YbgF, FkpA, Stx2B, OppA, DsbA, and SobB) or of unknown subcellular localization (five: Z1480, EspA, Z1483, Z1498, and Z3342; although EspA is a well-known extracellular protein). Clearly, most of these secreted proteins were reduced (blue) in the absence of cells and increased (red) in the presence of cells (Figure 6(C)).

Among the 49 differentially secreted proteins, 30 proteins in the absence of cells were differentially secreted among the mutants: in ΔT1SS 12 proteins (nine increased and three reduced), in ΔT2SS 10 proteins (two increased and eight reduced) and in ΔT3SS eight proteins (two increased and six reduced). Whereas, in the presence of cells, 47 proteins were differentially secreted: in ΔT1SS one protein (reduced), in ΔT2SS 21 proteins (2 increased, 19 reduced) and in ΔT3SS 25 proteins (16 increased and nine reduced). As most of these proteins could be reduced or increased at the same time in different mutants, in general, 28 out of the 49 proteins were differentially secreted in the presence of cells, in comparison to in the absence of cells, and 12 proteins were differentially secreted in the absence of cells vs in the presence of cells, and nine proteins were shared.

### Identification of abundant proteins in the supernatant of EHEC, ΔT1SS, ΔT2SS, and ΔT3SS

**Figure 7.**
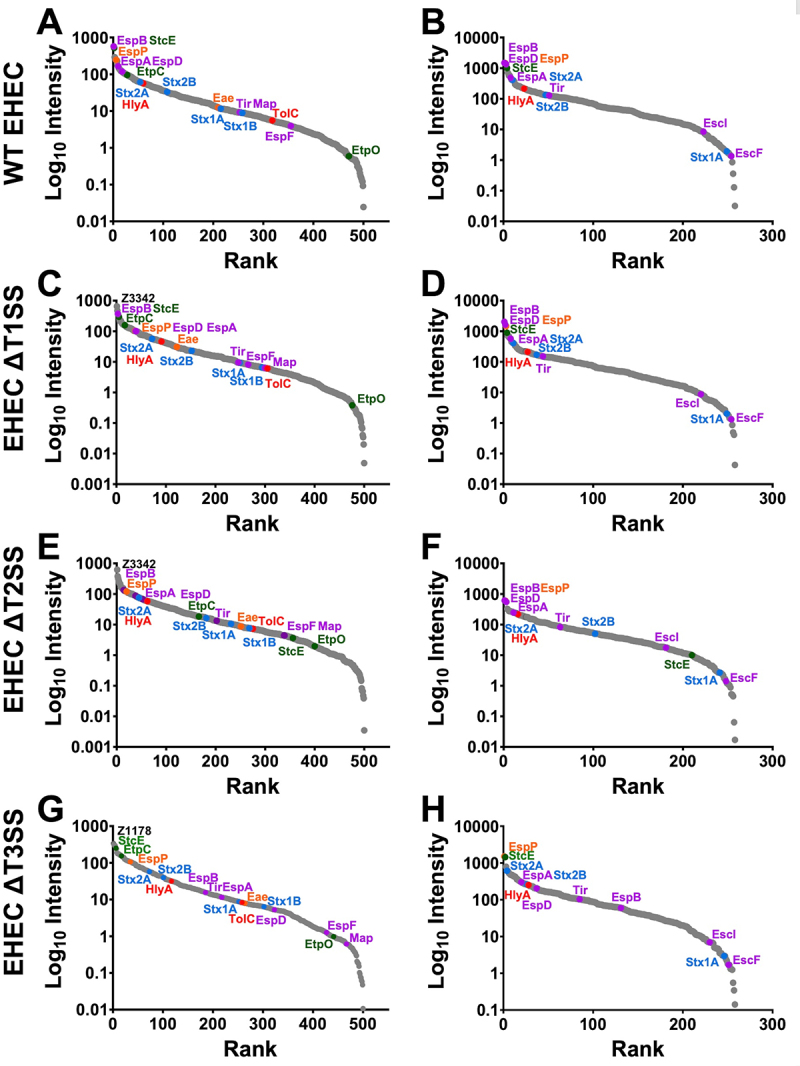
Proteomic analysis of protein abundance in the WT-EHEC, ΔT1SS, ΔT2SS or ΔT3SS secretomes in the absence or presence of epithelial cells. Rank plot showing protein abundance ordered by rank. WT-EHEC (A, B), ΔT1SS (C, D), ΔT2SS (E, F), or ΔT3SS (G, H) secretomes in the absence (A, C, E F) or presence (B, D, F H) of cells. Virulence factors have been highlighted: protein secreted by T1SS (red), T2SS (green), T3SS (magenta), T5SS (orange), Shiga toxins (sky blue), and other proteins (gray). Note the range of abundance of the highlighted virulence factor in the different mutants compared to the wild-type strain (in the presence of cells, the range on the y-axis is ten times greater).

To identify the abundance of the secreted proteins, WT-EHEC, ΔT1SS, ΔT2SS, or ΔT3SS supernatants were analyzed by quantitative mass spectrometry (LC-MS/MS), as mentioned above, followed by absolute label-free protein abundance estimation using intensity-based absolute quantification. The total number of proteins (500 in the absence of cells and 258 in the presence of cells) were quantified with different abundances, highlighting that in the presence of cells the overall secretion of proteins increased several times compared to in the absence of cells (Figure 7, note differences in the y-axis). The top 10 most abundant secreted proteins in the WT-EHEC supernatants in the absence of cells were EspB (NCS), StcE (SPI and NCS), UshA (SPI and NCS), Z3342 (NCS), GapA (NCS), EspP (SPI and NCS), Ssb (NCS), Pal (SPII and NCS), Z1178 (SPI and NCS), and EspA (NCS); all of them harbored a signal sequence. In addition, we performed an exhaustive search to construct a list of structural components and substrates of the SSs and Stx, which had been previously published (206 proteins in this database obtained from 50 papers). From this database, we highlighted the proteins classified in the rank plots. Thus, in the rank plot of WT-EHEC, the highlighted proteins in abundance rank were EspB, StcE, EspP, EspA, EspD, EtpC, Stx2A, HlyA, Stx2B, Eae, Stx1A, Tir, Map, Stx1B, TolC, EspF, and EtpO (Figure 7(A)). In the presence of cells, these profiles were changed. The top 10 proteins of highest abundance in WT-EHEC supernatants in the presence of cells were EspB (NCS), EspP (SPI and NCS), EspD (NCS), StcE (SPI and NCS), Gnd, PflB, TalB, EspA (NCS), Z1483 (NCS), and Stx2A (SPI); not all the proteins harbored a signal sequence; Gnd, PflB, and TalB lacked it. From our database, in the rank plot of WT-EHEC, the highlighted proteins in abundance rank were EspB, EspP, EspD, StcE, EspA, Stx2A, HlyA, Stx2B, Tir, EscI, Stx1A, and EscF (Figure 7(B)).

The top 10 most abundant proteins in ΔT1SS supernatants in the absence of cells were Z3342 (NCS), Z1178 (SPI and NCS), EspB (NCS), Ssb (NCS), StcE (SPI and NCS), YeaF (SPI and NCS), GapA (NCS), PurE, BamA (SPI and NCS), and PqqL (SPI and NCS); all of them harbored a signal sequence, except for PurE. From our database, in the rank plot of ΔT1SS, the highlighted proteins in abundance rank were EspB, StcE, EtpC, EspP, EspD, EspA, Stx2A, HlyA, Eae, Stx2B, Tir, Stx1A, EspF, Stx1B, Map, TolC, and EtpO (Figure 7(C)). While the top 10 most abundant proteins in the ΔT1SS supernatants in the presence of cells were EspB (NCS), EspD (NCS), EspP (SPI and NCS), StcE (SPI and NCS), PflB, TalB, Gnd, EspA (NCS), HNS, and Z1483 (NCS); not all the proteins harbored a signal sequence, PflB, TalB, Gnd, and HNS lacked it. From our database, in the rank plot of ΔT1SS the highlighted proteins in abundance rank were EspB, EspD, EspP, StcE, EspA, Stx2A, HlyA, Stx2B, Tir, EscI, Stx1A, and EscF (Figure 7(D)).

The top 10 most abundant proteins in ΔT2SS supernatants in the absence of cells were Z3342 (NCS), Ssb (NCS), Z1178 (SPI and NCS), YeaF (SPI and NCS), Eno, BamA (SPI and NCS), PurE, OppA (SPI and NCS), Pgk, and CcdA; Eno, PurE, Pgk, and CcdA lacked signal sequences. From our database, in the rank plot of ΔT2SS the highlighted proteins in abundance rank were EspB, EspP, EspA, Stx2A, EspD, HlyA, EtpC, Stx2B, Tir, Stx1A, Eae, Stx1B, TolC, EspF, Map, StcE, and EtpO (Figure 7(E)). While the top 10 most abundant proteins in the ΔT2SS supernatants in the presence of cells were EspB (NCS), EspP (SPI and NCS), EspD (NCS), Gnd, HNS, OmpA (SPI and NCS), Z1483 (NCS), Z1479 (NCS), PflB, and TalB; only Gnd, HNS, PflB, and TalB lacked signal sequences. From our database, in the rank plot of ΔT2SS, the highlighted proteins in abundance rank were EspB, EspP, EspD, EspA, Stx2A, HlyA, Tir, Stx2B, EscI, StcE, Stx1A, and EscF (Figure 7(F)).

The top 10 of the most abundant proteins in ΔT3SS supernatants in the absence of cells were Z1178 (SPI and NCS), UshA (SPI and NCS), Z3342 (NCS), Ssb (NCS), GapA (NCS), StcE (SPI and NCS), YeaF (SPI and NCS), Eno, Z1480 (NCS), and PurE; Eno and PurE lacked signal sequences. From our database, in the rank plot of ΔT3SS the highlighted proteins in abundance rank were StcE, EtpC, EspP, Stx2A, Stx2B, HlyA, EspB, EspA, Tir, Stx1A, TolC, Eae, Stx1B, EspD, EspF, EtpO, and Map but with different abundance that those seen in WT-EHEC (Figure 7(G)). Thus, for example EspB and EspA in ΔT3SS had a log10 intensity of 1.22 (number 185 in the abundance rank: 15.5 fmol) and 1.10 (217 in the rank: 11.5 fmol) vs 2.76 (1 in rank: 573.8 fmol) and 2.22 (10 in the rank: 165.6 fmol) in the WT-EHEC. While the top 10 most abundant proteins in the ΔT3SS supernatants in the presence of cells were EspP (SPI and NCS), StcE (SPI and NCS), Gnd, Stx2A (SPI), Ssb (NCS), Z1483 (NCS), HNS, OppA (SPI and NCS), SodB (NCS), and TpiA; only Gnd, HNS, and TpiA lacked a signal sequence. From our database, in the rank plot of ΔT3SS, the highlighted proteins in abundance rank were EspP, StcE, Stx2A, EspA, Stx2B, HlyA, EspD, Tir, EspB, EscI, Stx1A, and EscF (Figure 7(H)).

### Mutation in one SS affects the secretion of some effectors of other SSs

**Figure 8.**
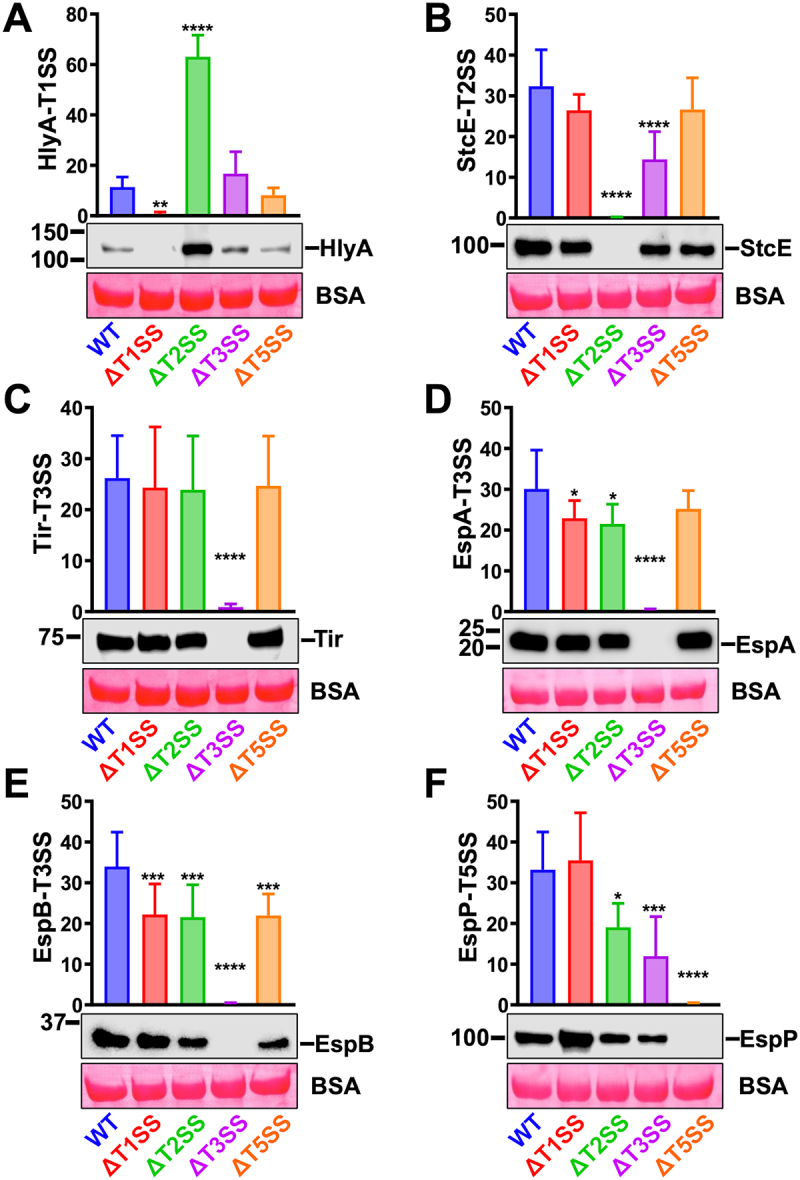
A mutation in one secretion system affects the secretion of some substrates of another secretion system. (A-F) WT-EHEC, ΔT1SS, ΔT2SS, ΔT3SS and ΔT5aSS were activated in MM9 during 4 h at 37°C and 5% CO_2_. Activated cultures were inoculated into Petri dishes with D_HG_, adjusting an amount of bacteria equivalent to a MOI of 10 in the absence of cells. After 3 h, supernatants were filtered and precipitated with 15% of TCA. An equivalent of 3 mL of supernatant was analyzed by Western blot using antibodies against substrates secreted by the T1SS (HlyA), T2SS (StcE), T3SS (Tir, EspB and EspA) and T5aSS (EspP). The plotted data show the mean ± SD from at least six independent experiments and a representative Western blot is shown. Statistical analyses were performed using one-way ANOVA followed by Dunnett’s test, comparing each treatment against WT-EHEC (*, *p* < 0.05; **, *p* < 0.01; ***, *p* < 0.001; ****, *p* < 0.0001).

To corroborate the possible interdependence between the SSs detected by proteomics, we compared the protein secretion of defined substrates of the different SSs of WT-EHEC vs ΔT1SS, ΔT2SS, ΔT3SS, and ΔT5SS by Western blot using antibodies directed against specific substrates. As expected, HlyA was not secreted in ΔT1SS supernatants in the absence of cells, and its secretion by ΔT3SS and ΔT5SS was similar to that of WT-EHEC. However, ΔT2SS caused a strong increase in the secretion of HlyA (T1SS substrate) (Figure 8(A)). On the contrary, StcE was not secreted by ΔT2SS as expected and was secreted at a similar level by WT-EHEC, ΔT1SS, and ΔT5SS; but significantly decreased in ΔT3SS (Figure 8(B)). In the case of the T3SS effectors (Figure 8(C-E)), Tir secretion in WT-EHEC was similar to all the mutants, except for ΔT3SS; EspA secretion was similar in WT-EHEC and ΔT5SS, but it was slightly decreased in ΔT1SS and ΔT2SS. The most dramatic case was for EspB, since its secretion was significantly decreased in all the mutants vs the WT-EHEC. While in the case of T5SS (Δ*espP*), ΔT1SS secreted similar levels of EspP than those of WT-EHEC, but the secretion of EspP decreased drastically in supernatants of ΔT3SS at a lower level than in ΔT2SS (Figure 8(F)).

**Figure 9.**
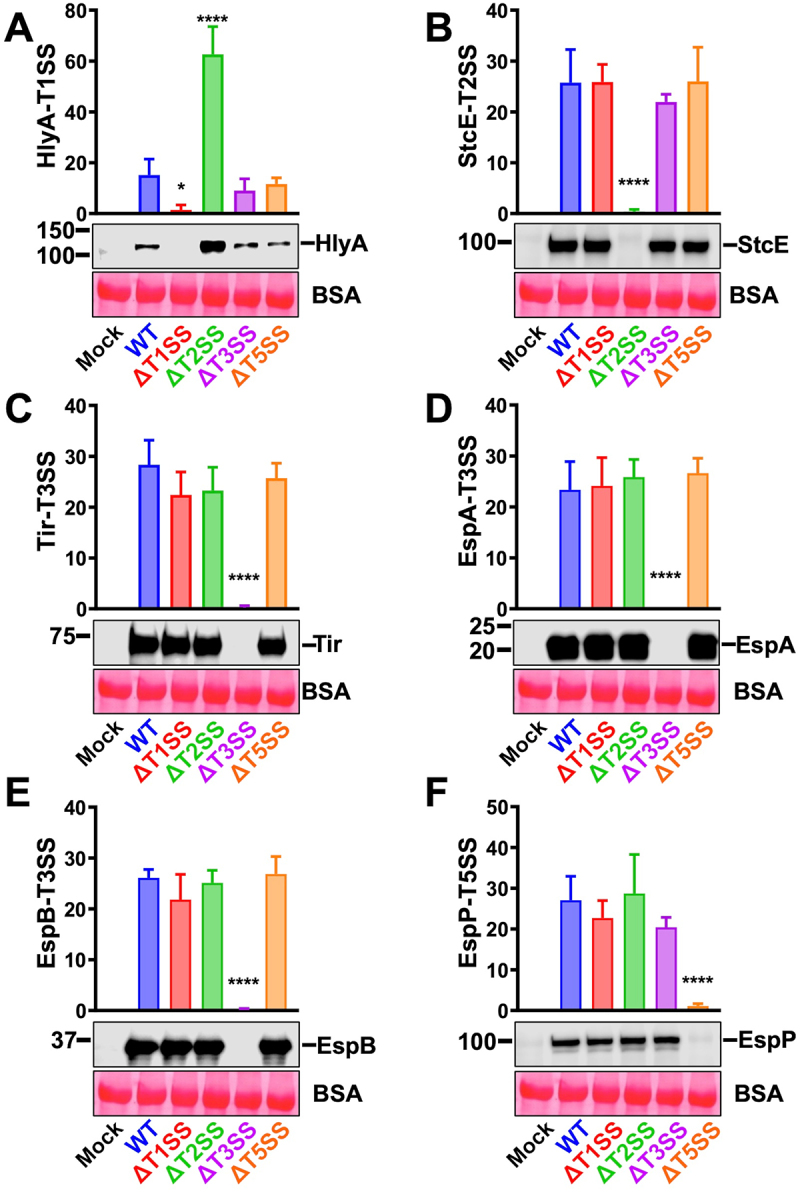
The presence of epithelial cells attenuates the effect caused by the absence of any EHEC secretion systems. (A) secretion of HlyA-T1SS, (B) StcE-T2SS, (C-E) Tir, EspA, EspB-T3SS, (F) EspP-T5SS. WT-EHEC, ΔT1SS, ΔT2SS, ΔT3SS, and ΔT5aSS were activated in MM9 during 4 h and subsequently inoculated into D_HG_. Supernatants were obtained after infecting HT-29 cells with WT-EHEC or its different mutants at MOI 10 during 3 h. The supernatants were filtered and precipitated with 15% of TCA. BSA was added as a loading control. An equivalent of 3 mL of precipitated supernatant was analyzed by Western blot. Densitometric analyses of the different secreted proteins were normalized with the final OD_600_ of each culture. The plotted data show the mean ± SD from four independent experiments and a representative Western blot is shown. Statistical analysis was performed using one-way ANOVA followed by a Dunnett’s test, comparing each treatment against WT-EHEC (*, *p* < 0.05; ****, *p <*0.0001).

Remarkably corroborating proteomics results, in the presence of cells, differences in StcE secretion (T2SS) or EspA, and EspB (T3SS) or EspP (T5aSS) were compensated in all mutants, and their secretion in the different mutants was similar to that in WT-EHEC (Figure 9(A-F)). However, there is still interdependence between T1SS and T2SS regarding HlyA secretion since the mutation in the T2SS caused a strong increase in HlyA secretion (T1SS substrate).

## Discussion

Several substrates are secreted by secretion systems, and these proteins achieve a function either in the extracellular medium or inside the eukaryotic cells [12–15]. Since the interaction between bacteria and eukaryotic cells requires multiple proteins that are secreted at a given time and space, understanding how EHEC integrates these different SSs, will allow us to determine their relevance during the infectious process. Here, we optimized the secretion of proteins for four of the five SSs of EHEC, since the T6SS is inactive under *in vitro* conditions [10]. We combined a medium such as MM9 to activate the SSs and DMEM medium for protein secretion into the extracellular medium. Remarkably, EHEC increases the secretion of specific proteins through different SSs in the presence of epithelial cells, many of which are specific virulence factors such as those detected by Western blot. Here, we also reported for the first time that the secretion of virulence factors through four EHEC SSs occurs via a secretion hierarchy during epithelial cell infection. Our analyses of the secretome of EHEC in the presence and absence of cells and its comparison with the different mutants in the SSs showed several important findings: EHEC substantially increases the abundance of specific substrates (virulence factors) in the presence of cells; the absence of a particular SS globally affects the secretion of substrates, and other substrates secreted by other SSs; the presence of epithelial cells, in some cases, drop the effects caused by the mutation of any SS seen in the absence of cells; bioinformatic analysis allowed us to identify new potential substrates for each SS. Validation analysis by Western blot showed that there is an interdependence for substrate secretion among the SSs.

EHEC strategically regulates the T3SS expression in response to its availability to oxygen and nutrients in the medium through the master regulator Ler [38,39]. Here, we showed that the secretion of substrates associated to four SSs (HlyA, StcE, Tir, EspB, EspA, and EspP) was improved in a medium with minimum salts and low glucose (MM9) to activate EHEC for protein secretion. Additionally, after activation, it was possible to optimize the substrate secretion by incubating EHEC-infected cells in a medium for eukaryotic cells. Thereby, the best compromise for protein secretion and epithelial cell preservation during the experiment was using MM9 for activation and DMEM high glucose during the secretion experiments in the presence or absence of epithelial cells. In the lack of calcium in the M9 medium, EHEC mainly secretes effectors, such a Tir and NleA, whereas DMEM low glucose with pyruvate increases the LEE gene transcription and the secretion of the translocator EspB [27,39]. Interestingly, if EHEC is kept in MM9 medium all the time (activation and secretion), the secretion of substrates by T2SS (StcE) and T3SS (Tir, EspB, and EspA) was substantially increased as compared to DMEM low/high glucose, which positions our MM9 as the best medium to study the secretion of these two SSs. In DMEM low glucose, HlyA secretion was improved compared to DMEM high glucose, traditionally used for protein secretion by T3SS. In concordance with the fact that Ler transcription is activated in low glucose media [38]. In addition, Ler not only positively regulates the T3SS expression but also promotes the expression of HlyA-T1SS and StcE-T2SS [13,40]. Unlike T3SS, T1SS does not function efficiently in MM9 medium, suggesting that T1SS requires low glucose levels and nutrients present in the DMEM medium to function efficiently. The MM9 medium, formulated in this work, contains pyruvate as a carbon source alternative to glucose. This medium not only optimized the secretion by T3SS but also by T2SS. This agrees with a recent report showing that PdhR, the master regulator of carbon metabolism, is a LEE activator that responses to pyruvate levels in the medium [41]. Since Ler also promotes the T2SS expression, it is quite possible that these two SSs could be working in a coordinated fashion during the infectious process.

Our proteomics data showed that EHEC secretes proteins through their four different SS either in the absence or presence of eukaryotic cells. However, when epithelial cells are infected with EHEC, the increase in specific substrate secretion through T1SS, T2SS, T3SS, and T5SS is higher, in spite that the overall number of secreted proteins is reduced. These data were consistent with an increase in the secretion of specific substrates for each SS detected by Western blot. These findings are interesting because the proteins that are inserted into the host cell membrane (e.g. Tir) or translocate into cells (e.g. EspB) could be even more increased and therefore underestimated in this study. This strong increase in protein secretion in the presence of host cells could be related to previous reports showing that components of the plasma membrane of eukaryotic cells contribute to pathogenesis, as reported for the effectors of T3SS of *Salmonella*, *Shigella*, and EPEC [42,43]. Eukaryotic cell supernatants did not appear to modify the secreted bacterial effectors at 3 h of interaction (data not shown). On the other hand, host cell sphingolipids are mediators of pathogenesis throughout T3SS and the Shiga toxin [44]. These data suggest that EHEC could sense components of the host membrane to stimulate protein secretion by the four different SSs. This idea is relevant and supports the existence of high cellular communications between these two kingdoms, such as epinephrine/norepinephrine regulation [45,46].

Through comparative analyses of secreted proteins in the supernatants by proteomics and using the WT-EHEC and mutants in T1SS, T2SS and T3SS, in the absence or presence of cells, we found that the lack of any SS produced a global change in the EHEC secretion profile. Interestingly, in the absence of cells, ΔT3SS shares the smaller percentage (85%) of protein secreted in common with WT-EHEC, while ΔT1SS and ΔT2SS shared 91% and 95%. This marked difference in the secretion profile in ΔT3SS could explain why a mutation in this SS abolishes almost all morphological effects on the epithelial cells [47]. Additionally, EHEC harbors a wider repertory of secreted effectors (39) [11], and consistently, in the presence of cells, ΔT3SS secreted a larger number of proteins compared to WT-EHEC but the substrate profile was different. Interestingly, only one substrate (HlyA) has been experimentally reported to be secreted by T1SS [12], and 95% of protein secretion was shared between ΔT1SS and WT-EHEC, suggesting that EHEC harbors a few numbers of substrates secreted by T1SS. Although in ΔT2SS, 91% of the proteins identified in the supernatant were shared with WT-EHEC, fewer proteins (45) were identified in its supernatants. Contrastingly, in EHEC only two proteins have been experimentally found in its supernatants, StcE [13,16] and YodA [17], associated to mucin degradation and EHEC adherence to epithelial cells, respectively. However, T2SS is conserved among A/E pathogens, such as EHEC, EPEC, and *C. rodentium*. Again, in EPEC only one substrate has been identified, SslE lipoprotein, which is essential for biofilm formation [21]. But 22 substrates were identified by bioinformatic analyses in *C. rodentium* T2SS, which are functional and key for colonization [22]. We therefore speculate that additional substrates currently unknown in EHEC may be secreted through T2SS. However, T2SS has been poorly studied to date. ΔT3SS supernatants showed a decreased secretion of EspB, EspD, EspA, and Map. Tir and EspF were also identified, but they did not show significant differences compared to WT-EHEC supernatants. Therefore, our medium formulation (MM9) could be promoting the translocator secretion, since EPEC and *C. rodentium* secretomes can be modified by mutants in *sepD* and *sepL* (effector hypersecretion), whereas the classic DMEM medium used preferentially promotes translocator secretion [48,49]. The proteomics data were consistent with those obtained by Western blot, showing that ΔT3SS did not secrete EspB, EspA, and Tir. Furthermore, we confirmed that this mutant caused a decrease in EspP secretion (T5aSS) and, surprisingly, in StcE secretion (T2SS), but it was not detected by proteomics. Nevertheless, these findings support our hypothesis about the interdependence between T2SS, T3SS, and T5SS.

In the proteomics data, no significant difference in HlyA secretion was observed between ΔT1SS and WT-EHEC, which was unexpected. However, EHEC is known to produce outer membrane vesicles (OMVs) containing HlyA [50]. As expected, no HlyA secretion was detected by Western blot, in these assays the supernatant proteins were precipitated with trichloroacetic acid, whereas the supernatants analyzed by proteomics were concentrated by filtration. In contrast, when comparing the secretomes of WT-EHEC and ΔT2SS, StcE dramatically decreased both in the absence and presence of epithelial cells. Notably, the lack of T2SS altered the secretion of T3SS substrates, such as EspB and Map in the absence of cells, and EspB and EspA in the presence of cells. ΔT2SS also affected the presence of Stx2B in the supernatant in the presence of cells and EspP in the absence and presence of cells, both reaching the periplasm. These data were consistent with those found by Western blot; ΔT2SS altered EspB, EspA, and EspP secretions. However, in the presence of cells, these differences become not significant, except for StcE. Thus, besides the relevance of Ler as a positive regulator of StcE-T2SS secretion [13,51], our data report the relevant involvement of T2SS in the secretion of substrates by T3SS. We also observed a substantial increase in HlyA-T1SS secretion in ΔT2SS supernatants in the absence and presence of cells by Western blot. HlyA expression is inhibited by HNS, a global regulator of various virulence factors in EHEC [8] and its regulation is also associated with the LEE island, such as Ler and GrlA [13]. However, the increased secretion of HlyA in ΔT2SS could be associated with the StcE secretion process, since the StcE protein is first translocated to the periplasm (by its SP) where it becomes arrested due to the absence of T2SS (Δ*etpC*). This accumulation of StcE metalloprotease could produce stress in the bacterial membrane, which would activate the sigma stress factor RpoS, essential for HlyA expression [52]. This result was not corroborated by proteomics analyses, but it is explained by the difference in the protein concentration methods that could exclude OMVs.

The use of mutants in T1SS, T2SS, and T3SS in combination with several selected features allowed us to find new candidate substrates. The secreted proteins must harbor an export sequence (SP or/and NCS), with subcellular localization no related to the cytoplasm, not associated previously to a SS and having a prediction as putative substrates by BastionHub [33–36,53]. Most of the proteins found were 13 putative T2SS substrates, followed by a few T3SS substrate candidates (three proteins) and only one of T1SS. Remarkably, most of the candidates are harbored in bacteriophage sequences and more important, they are encoded in pathogenicity islands associated to virulence (around 15 proteins), nearby other virulence factors mainly Nle genes and varieties of Shiga toxin genes (Table 3). It is likely that these candidates could be important virulence factors that could contribute to the pathogenesis of EHEC.

Different studies have shown that EHEC uses different SSs during the infection [13,53–56]. Therefore, it is important to know how these SSs are used by EHEC when it faces an epithelial cell. Our data showed that EHEC follows a secretion hierarchy for protein secretion throughout its four SSs. EspA-T3SS was the first most abundant protein secreted, possibly for the high number of EspA monomers required to form the EspA filament. In contrast, the EspB translocator was detected after the secretion of EspA and Tir, suggesting that once the filament begins its formation, Tir might be secreted or perhaps the amount of EspB needed to form the pore in the host membrane is less than that of Tir to be detected before. Anyway, these data are consistent with findings showing that SepD and SepL proteins regulate the secretion hierarchy between translocators and effectors [27]. While CesT and CesL chaperones stabilize the effectors into the bacterial cytoplasm and target them for translocation by T3SS, thus modulating the translocation efficiency [57]. Thus, Tir is one of the first effectors secreted in the presence of the host cell, demonstrating its importance for the intimate adhesion of EHEC to the host [58]. Surprisingly, T1SS was the second SS to be activated as detected by HlyA secretion. Although it has been suggested that HlyA secretion in EHEC is deficient, leading to the use of inducible vectors for its expression [59], here we have found culture conditions that will be helpful to study the protein secretion by T1SS. Finally, we detected simultaneous secretion by T2SS and T5aSS. Interestingly, in the presence of cells, the secretion of StcE and EspP was detected at earlier times (40 vs 60 min in the absence of cells); this could be related to the effect that epithelial cells have on stimulating protein secretion. However, the secretion hierarchy by the different SSs is conserved in both the absence and presence of cells. It is worth mentioning that, despite the high energy demand required to express all these protein complexes, EHEC can secrete substrates (virulence factors) into the extracellular medium by four different SSs, among them three nanomachines. This fact might explain the success of EHEC in carrying out colonization from a low infectious dose.

## Supplementary Material

Supplemental Table S1 to S13.docx

## Data Availability

The authors confirm that the data supporting the findings of this study are available within the article and its supplementary materials. The data that support the findings of this study are available in PRIDE Archive (mass spectrometry proteomics data) at http://doi.org/10.6019/PXD067036 and http://doi.org/10.6019/PXD067077, reference number P×D067036 (EHEC secretome in absence of epithelial cells) and P×D067077 (in presence of epithelial cells).
