# Supplementary material for: Substrate secretion by different EHEC secretion systems during their interaction with epithelial cells
Source: Virulence. 2026 Feb 19;17(1):2634461. doi: 10.1080/21505594.2026.2634461 (PMC12962677; doi:10.1080/21505594.2026.2634461)
Supplement: Supplemental Table S1 to S13.docx [file KVIR_A_2634461_SM8336.docx]

**Table S1. Top ten proteins with the highest fold change in EHEC ΔT1SS in the absence of cells**

| **Accesion** | **Name** | **Peptide count** | **Unique peptides** | **Fold Change (Log2)** | | | ***Q value* (-Log10)** | | | **SignalP** | **SecretomeP** | **PSORTb** |
| --- | --- | --- | --- | --- | --- | --- | --- | --- | --- | --- | --- | --- |
|  |  |  |  | **ΔT1SS** | **ΔT2SS** | **ΔT3SS** | **ΔT1SS** | **ΔT2SS** | **ΔT3SS** |  |  |  |
| **Increased** | | | | | | | | | | | | |
| P0AAD0 | IscA | 3 | 1 | 4.6 | 3.5 | 2.2 | 3.0 | 2.0 | 2.1 | OTHER | NCS | Cytoplasmic |
| P0AEA0 | CsgF | 2 | 1 | 4.5 | 1.3 | 1.1 | 3.5 | 2.6 | 1.9 | SPI | NCS | Extracellular |
| P0AB08 | YcbK | 6 | 2 | 4.2 | 2.5 | 2.2 | 4.1 | 3.0 | 1.3 | SPI-Tat | None | Unknown |
| A0A5Q2EJ51 | Z5646 | 12 | 5 | 3.6 | 1.6 | 0.7 | 2.7 | 2.9 | 0.9 | OTHER | None | Cytoplasmic |
| Q8X7W7 | Fiu | 46 | 22 | 3.3 | -0.5 | 1.3 | 3.9 | 0.7 | 1.6 | SPI | NCS | Outer Membrane |
| P0ACG0 | HNS | 10 | 7 | 3.1 | 0.7 | 1.5 | 2.8 | 1.7 | 1.3 | OTHER | None | Cytoplasmic |
| P0ABA8 | AtpG | 6 | 1 | 3.0 | 1.7 | 1.3 | 2.3 | 2.2 | 1.9 | OTHER | None | Cytoplasmic Membrane |
| A0A4P8B5E5 | BamB | 18 | 14 | 2.7 | 1.6 | 2.4 | 2.5 | 1.1 | 0.6 | SPII | NCS | Outer Membrane |
| A0A5Q2ETC9 | VacJ | 8 | 4 | 2.6 | 1.4 | 1.3 | 2.9 | 3.2 | 1.5 | SPII | NCS | Outer Membrane |
| Q8XDE6 | PepN | 62 | 43 | 2.5 | 0.6 | 0.9 | 4.1 | 3.0 | 2.7 | OTHER | None | Cytoplasmic |
| **Reduced** | | | | | | | | | | | | |
| Q8XCI7 | AspS | 13 | 8 | -2.4 | -2.1 | -1.3 | 2.6 | 2.2 | 2.0 | OTHER | None | Cytoplasmic |
| A0A6M7H4H9 | TbpA | 5 | 3 | -1.7 | -1.3 | -1.8 | 2.2 | 1.9 | 2.0 | SPI | NCS | Periplasmic |
| A0A4P8BD36 | Z1482 | 8 | 2 | -1.6 | -0.4 | 0.3 | 2.9 | 1.4 | 0.5 | OTHER | None | Cytoplasmic |
| P68067 | GrcA | 10 | 6 | -1.5 | -2.0 | -2.5 | 2.5 | 2.2 | 2.3 | OTHER | None | Cytoplasmic |
| P0A7T9 | RpsR | 10 | 8 | -1.5 | -1.0 | -1.8 | 2.3 | 1.8 | 2.1 | OTHER | None | Cytoplasmic |
| P0A7J9 | RplK | 8 | 7 | -1.5 | -1.6 | -1.8 | 2.8 | 2.7 | 2.4 | OTHER | NCS | Cytoplasmic |
| P0A736 | MinE | 1 | 1 | -1.4 | -1.6 | -2.3 | 2.5 | 2.3 | 2.4 | OTHER | None | Cytoplasmic |
| P0AG53 | RpmD | 7 | 6 | -1.4 | -2.0 | -1.8 | 2.4 | 2.3 | 2.1 | OTHER | None | Cytoplasmic |
| P0ABB6 | AtpD | 16 | 8 | -1.3 | -3.0 | -1.5 | 2.6 | 2.9 | 2.3 | OTHER | None | Cytoplasmic Membrane |
| A0A4P8BDD0 | Z1432 | 3 | 2 | -1.3 | -0.5 | -2.9 | 2.6 | 1.1 | 2.5 | OTHER | None | Cytoplasmic |

**Table S2. Top ten proteins with highest *Q-value* in EHEC ΔT1SS in the absence of cells**

| **Accesion** | **Name** | **Peptide count** | **Unique peptides** | **Fold Change (Log2)** | | | ***Q value* (-Log10)** | | | **SignalP** | **SecretomeP** | **PSORTb** |
| --- | --- | --- | --- | --- | --- | --- | --- | --- | --- | --- | --- | --- |
|  |  |  |  | **ΔT1SS** | **ΔT2SS** | **ΔT3SS** | **ΔT1SS** | **ΔT2SS** | **ΔT3SS** |  |  |  |
| **Increased** | | | | | | | | | | | | |
| Q8XDE6 | PepN | 62 | 43 | 2.5 | 0.6 | 0.9 | 4.1 | 3.0 | 2.7 | OTHER | None | Cytoplasmic |
| P0AB08 | YcbK | 6 | 2 | 4.2 | 2.5 | 2.2 | 4.1 | 3.0 | 1.3 | SPI-Tat | None | Unknown |
| P67911 | HldD | 21 | 12 | 2.2 | 1.1 | -0.1 | 3.9 | 3.2 | 0.3 | OTHER | None | Cytoplasmic |
| Q8X9G6 | Pgm | 34 | 17 | 1.6 | 0.3 | 0.0 | 3.9 | 1.1 | 0.2 | OTHER | None | Cytoplasmic |
| Q8X7W7 | Fiu | 46 | 22 | 3.3 | -0.5 | 1.3 | 3.9 | 0.7 | 1.6 | SPI | NCS | Outer Membrane |
| A0A6M7H3L5 | ChuT | 17 | 13 | 2.5 | 0.7 | 0.6 | 3.6 | 2.1 | 2.5 | OTHER | None | Periplasmic |
| P0A760 | NagB | 9 | 4 | 2.4 | 0.3 | 0.6 | 3.5 | 0.5 | 0.7 | OTHER | None | Cytoplasmic |
| P0AEA0 | CsgF | 2 | 1 | 4.5 | 1.3 | 1.1 | 3.5 | 2.6 | 1.9 | SPI | NCS | Extracellular |
| P58256 | IlvC | 52 | 30 | 1.4 | -0.4 | -0.6 | 3.5 | 1.4 | 1.6 | OTHER | None | Cytoplasmic |
| P0A7D6 | PurA | 33 | 14 | 2.0 | -0.4 | -0.1 | 3.3 | 0.9 | 0.2 | OTHER | None | Cytoplasmic |
| **Reduced** | | | | | | | | | | | | |
| Q7BSW5 | EspP | 274 | 193 | -1.3 | -1.0 | -1.2 | 3.0 | 3.2 | 2.3 | SPI | NCS | Extracellular |
| A0A5Q2EU73 | PotD | 41 | 24 | -1.2 | -2.2 | -1.1 | 3.0 | 3.2 | 2.4 | SPI | NCS | Periplasmic |
| A0A4P8BD36 | Z1482 | 8 | 2 | -1.6 | -0.4 | 0.3 | 2.9 | 1.4 | 0.5 | OTHER | None | Cytoplasmic |
| P0A7J9 | RplK | 8 | 7 | -1.5 | -1.6 | -1.8 | 2.8 | 2.7 | 2.4 | OTHER | NCS | Cytoplasmic |
| A0A4P8BDD0 | Z1432 | 3 | 2 | -1.3 | -0.5 | -2.9 | 2.6 | 1.1 | 2.5 | OTHER | None | Cytoplasmic |
| P0AFG7 | SucB | 22 | 12 | -1.1 | -0.1 | -0.4 | 2.6 | 0.6 | 1.5 | OTHER | None | Cytoplasmic |
| Q8XCI7 | AspS | 13 | 8 | -2.4 | -2.1 | -1.3 | 2.6 | 2.2 | 2.0 | OTHER | None | Cytoplasmic |
| P0ABB6 | AtpD | 16 | 8 | -1.3 | -3.0 | -1.5 | 2.6 | 2.9 | 2.3 | OTHER | None | Cytoplasmic Membrane |
| P0A736 | MinE | 1 | 1 | -1.4 | -1.6 | -2.3 | 2.5 | 2.3 | 2.4 | OTHER | None | Cytoplasmic |
| P68067 | GrcA | 10 | 6 | -1.5 | -2.0 | -2.5 | 2.5 | 2.2 | 2.3 | OTHER | None | Cytoplasmic |

**Table S3. Differentially secreted proteins with signal sequence identify in supernatants in the absence of cells**

| **ID** | **Name** | **SignalP** | **SecretomeP** | **PSORTb** | **Secretion System** | **Differentially secreted** | | |
| --- | --- | --- | --- | --- | --- | --- | --- | --- |
|  |  |  |  |  |  | **ΔT1SS** | **ΔT2SS** | **ΔT3SS** |
| Q8XCI0 | FklB | SPI | None | Cytoplasmic | Unknown | NS | Down | NS |
| A0A4P8BAV1 | Z1498 | SPI | None | Unknown | Unknown | NS | Down | NS |
| P0AB08 | YcbK | SPI-Tat | None | Unknown | Unknown | Up | Up | NS |
| A0A5Q2ETC9 | VacJ | SPII | NCS | Outer Membrane | Unknown | Up | Up | NS |
| A0A6M7H5S1 | YbhC | SPII | NCS | Outer Membrane | Unknown | Up | NS | NS |
| A0A4P8B5E5 | BamB | SPII | NCS | Outer Membrane | Unknown | Up | NS | NS |
| A0A0H3JHG0 | NlpB | SPII | NCS | Outer Membrane | Unknown | Up | NS | Up |
| P0A913 | Pal | SPII | NCS | Outer Membrane | Unknown | NS | Down | NS |
| P61321 | LolB | SPII | NCS | Periplasmic | Unknown | Up | NS | NS |
| Q8XAV7 | PqqL | SPI | NCS | Cytoplasmic Membrane | Unknown | Up | NS | NS |
| P0AEA0 | CsgF | SPI | NCS | Extracellular | Unknown | Up | Up | NS |
| A0A5Q2EQN3 | Z2200 | SPI | NCS | Extracellular | Unknown | Up | Up | NS |
| O82882 | StcE | SPI | NCS | Extracellular | T2SS | NS | Down | NS |
| Q8XA13 | LptD | SPI | NCS | Outer Membrane | Unknown | Up | NS | NS |
| Q8X8U8 | Z3159 | SPI | NCS | Outer Membrane | Unknown | Up | NS | NS |
| A0A4P8B8F8 | Z2239 | SPI | NCS | Outer Membrane | Unknown | Up | NS | NS |
| Q8X7W7 | Fiu | SPI | NCS | Outer Membrane | Unknown | Up | NS | NS |
| P43261 | Eae | SPI | NCS | Outer Membrane | T5eSS | Up | NS | NS |
| P0A942 | BamA | SPI | NCS | Outer Membrane | Unknown | Up | Up | NS |
| A0A4P8BC04 | Z1178 | SPI | NCS | Outer Membrane | Unknown | Up | NS | NS |
| Q7BSW5 | EspP | SPI | NCS | Outer Membrane | T5aSS | Down | Down | Down |
| A0A4P8B2K4 | ChuA | SPI | NCS | Outer Membrane | Unknown | NS | Up | NS |
| P0ADS7 | YggE | SPI | NCS | Periplasmic | Unknown | Up | NS | NS |
| A0A5Q2EPE0 | AfuA | SPI | NCS | Periplasmic | Unknown | Up | NS | NS |
| A0A6M7GZ14 | PotF | SPI | NCS | Periplasmic | Unknown | Up | NS | NS |
| A0A6M7H2U1 | YbgF | SPI | NCS | Periplasmic | Unknown | Up | Up | NS |
| A0A6M7H4H9 | TbpA | SPI | NCS | Periplasmic | Unknown | Down | NS | Down |
| A0A5Q2EU73 | PotD | SPI | NCS | Periplasmic | Unknown | Down | Down | Down |
| P61318 | LolA | SPI | NCS | Periplasmic | Unknown | NS | Up | NS |
| A0A5Q2EPR3 | CpdB | SPI | NCS | Periplasmic | Unknown | NS | Down | NS |
| A0A6M7GWT5 | MglB | SPI | NCS | Periplasmic | Unknown | NS | Down | Down |
| A0A6M7H3H7 | YtfJ | SPI | NCS | Unknown | Unknown | Up | NS | NS |
| A0A6M7GZR0 | Z2206 | SPI | NCS | Unknown | Unknown | Up | Up | NS |
| Q8XB75 | HiuH | SPI | NCS | Unknown | Unknown | Up | NS | NS |
| P0AAA4 | EcpA | SPI | NCS | Unknown | Unknown | NS | Up | NS |
| P0A8X3 | YceI | SPI | NCS | Unknown | Unknown | NS | Up | Up |
| P0AAD0 | IscA | OTHER | NCS | Cytoplasmic | Unknown | Up | NS | Up |
| P0A7J9 | RplK | OTHER | NCS | Cytoplasmic | Unknown | Down | Down | Down |
| P0A7U5 | RpsS | OTHER | NCS | Cytoplasmic | Unknown | NS | Up | NS |
| P0A7S1 | RpsK | OTHER | NCS | Cytoplasmic | Unknown | NS | Up | NS |
| P0A6N6 | Efp | OTHER | NCS | Cytoplasmic | Unknown | NS | Down | Down |
| P0A9Y8 | CspC | OTHER | NCS | Cytoplasmic | Unknown | NS | NS | Down |
| A0A4P8B4C9 | EspF | OTHER | NCS | Extracellular | T3SS-LEE | Up | NS | NS |
| Q7DBI0 | FliC | OTHER | NCS | Extracellular | Unknown | Down | NS | NS |
| A0A6M7GT08 | EspB | OTHER | NCS | Extracellular | T3SS-LEE | NS | Down | Down |
| Q8X3W0 | Z0982 | OTHER | NCS | Extracellular | Unknown | NS | NS | Up |
| A0A4P8B4A7 | EspD | OTHER | NCS | Extracellular | T3SS-LEE | NS | NS | Down |
| Q8XB86 | Z3058 | OTHER | NCS | Outer Membrane | Unknown | Up | NS | NS |
| A0A6M7GZ37 | ManA | OTHER | NCS | Periplasmic | Unknown | NS | Up | NS |
| Q8X944 | GalM | OTHER | NCS | Periplasmic | Unknown | NS | Up | NS |
| A0A4P8B641 | Z3087 | OTHER | NCS | Unknown | Unknown | Up | NS | NS |
| A0A384L4N7 | Z2137 | OTHER | NCS | Unknown | Unknown | Up | NS | NS |
| A0A0H3JIZ8 | Z2442 | OTHER | NCS | Unknown | Unknown | Up | Up | NS |
| A0A6M6XA91 | Z1483 | OTHER | NCS | Unknown | Unknown | Up | NS | NS |
| A0A6M6W3H8 | Z1430 | OTHER | NCS | Unknown | Unknown | NS | Up | NS |
| A0A6M7GSY4 | Map | OTHER | NCS | Unknown | T3SS-LEE | NS | Down | Down |
| A0A5Q2EW70 | EspA | OTHER | NCS | Unknown | T3SS-LEE | NS | NS | Down |

**Table S4. Differentially secreted proteins with signal sequence identify in supernatants in the presence of cells**

| **ID** | **Name** | **SignalP** | **SecretomeP** | **PSORTb** | **Secretion System** | **Differentially secreted** | | |
| --- | --- | --- | --- | --- | --- | --- | --- | --- |
|  |  |  |  |  |  | **ΔT1SS** | **ΔT2SS** | **ΔT3SS** |
| A0A6M7H7A3 | Z0372 | SPI | None | Unknown | Unknown | NS | Down | Up |
| A0A4P8BAV1 | Z1498 | SPI | None | Unknown | Unknown | NS | NS | Up |
| A0A4V1DN87 | Z2371 | SPI | None | Unknown | Unknown | NS | NS | Up |
| A0A0H3JL10 | Slp | SPII | NCS | Outer Membrane | Unknown | NS | Down | NS |
| P0A913 | Pal | SPII | NCS | Outer Membrane | Unknown | NS | Down | NS |
| A0A6M6W7P0 | BorW | SPII | NCS | Unknown | Unknown | NS | Down | NS |
| O82882 | StcE | SPI | NCS | Extracellular | T2SS | NS | Down | NS |
| Q7BSW5 | EspP | SPI | NCS | Outer Membrane | T5aSS | NS | Down | NS |
| A0A4P8BAU8 | Stx2B | SPI | NCS | Periplasmic | Stx | NS | Down | NS |
| Q8XDA4 | OppA | SPI | NCS | Periplasmic | Unknown | NS | Down | NS |
| Q8X3U3 | YehC | SPI | NCS | Periplasmic | Unknown | NS | Down | Down |
| Q8XB75 | HiuH | SPI | NCS | Unknown | Unknown | NS | Down | NS |
| A0A0H3JKM0 | YhcN | SPI | NCS | Unknown | Unknown | NS | Down | NS |
| A0A4P8B779 | YeaF | SPI | NCS | Outer Membrane | Unknown | NS | NS | Up |
| P0AEU9 | Skp | SPI | NCS | Periplasmic | Unknown | NS | NS | Up |
| P0AEG5 | DsbA | SPI | NCS | Periplasmic | Unknown | NS | NS | Up |
| A0A6M7GSJ8 | AnsB | SPI | NCS | Periplasmic | Unknown | NS | NS | Up |
| A0A6M6S3U5 | Z2099 | SPI | NCS | Unknown | Unknown | NS | NS | Up |
| A0A4P8B947 | Z2967 | SPI | NCS | Unknown | Unknown | NS | NS | Up |
| A0A0H3JGK4 | Z3508 | SPI | NCS | Unknown | Unknown | NS | NS | Up |
| A0A4P8BC04 | Z1178 | SPI | NCS | Outer Membrane | Unknown | NS | NS | Down |
| P65765 | FkpA | SPI | NCS | Periplasmic | Unknown | NS | NS | Down |
| P0A7M4 | RpmB | OTHER | NCS | Cytoplasmic | Unknown | NS | Up | NS |
| P58217 | Z2083 | OTHER | NCS | Unknown | Unknown | NS | Up | NS |
| P60440 | RplC | OTHER | NCS | Cytoplasmic | Unknown | NS | Down | NS |
| P0A9B4 | GapA | OTHER | NCS | Cytoplasmic | Unknown | NS | Down | NS |
| P0A9Y1 | CspA | OTHER | NCS | Cytoplasmic | Unknown | NS | Down | NS |
| A0A4P8B4A7 | EspD | OTHER | NCS | Extracellular | T3SS-LEE | NS | Down | Down |
| A0A6M7GT08 | EspB | OTHER | NCS | Extracellular | T3SS-LEE | NS | Down | Down |
| Q8X2B4 | Z1466 | OTHER | NCS | Unknown | Unknown | NS | Down | NS |
| A0A5Q2EPV8 | Z5884 | OTHER | NCS | Unknown | Unknown | NS | Down | NS |
| P0ACF6 | HupB | OTHER | NCS | Cytoplasmic | Unknown | NS | NS | Up |
| P0A9Y8 | CspC | OTHER | NCS | Cytoplasmic | Unknown | NS | NS | Up |
| P0AGE2 | Ssb | OTHER | NCS | Cytoplasmic | Unknown | NS | NS | Up |
| Q7DBI0 | FliC | OTHER | NCS | Extracellular | Unknown | NS | NS | Up |
| P0AGD5 | SodB | OTHER | NCS | Periplasmic | Unknown | NS | NS | Up |
| A0A6M6W3H8 | Z1430 | OTHER | NCS | Unknown | Unknown | NS | NS | Up |
| P0ACZ1 | YebG | OTHER | NCS | Unknown | Unknown | NS | NS | Up |
| Q8X5A7 | Z3074 | OTHER | NCS | Extracellular | Unknown | NS | NS | Down |

**Table S5. Top ten proteins with highest fold change in EHEC ΔT2SS in the absence of cells**

| **Accesion** | **Name** | **Peptide count** | **Unique peptides** | **Fold Change (Log2)** | | | ***Q value* (-Log10)** | | | **SignalP** | **SecretomeP** | **PSORTb** |
| --- | --- | --- | --- | --- | --- | --- | --- | --- | --- | --- | --- | --- |
|  |  |  |  | **ΔT1SS** | **ΔT2SS** | **ΔT3SS** | **ΔT1SS** | **ΔT2SS** | **ΔT3SS** |  |  |  |
| **Increased** | | | | | | | | | | | | |
| A0A4P8B4J6 | Z5009 | 8 | 4 | 0.7 | 2.5 | 0.8 | 1.3 | 3.2 | 0.8 | OTHER | None | Cytoplasmic |
| P0AB08 | YcbK | 6 | 2 | 4.2 | 2.5 | 2.2 | 4.1 | 3.0 | 1.3 | SPI-Tat | None | Unknown |
| Q8XEA1 | RdgC | 14 | 9 | -1.0 | 2.3 | -0.8 | 1.4 | 2.5 | 1.0 | OTHER | None | Cytoplasmic |
| A0A0H3JIZ8 | Z2442 | 8 | 6 | 1.6 | 1.9 | 2.0 | 2.5 | 2.9 | 1.4 | OTHER | NCS | Unknown |
| A0A5Q2ESY8 | Gmd1 | 15 | 10 | 1.4 | 1.8 | 1.0 | 1.7 | 2.0 | 0.9 | OTHER | None | Cytoplasmic |
| P58473 | PepB | 48 | 31 | 1.2 | 1.8 | 0.9 | 1.8 | 2.1 | 0.8 | OTHER | None | Cytoplasmic |
| P0A7S1 | RpsK | 16 | 11 | 0.1 | 1.7 | -0.6 | 0.4 | 3.0 | 0.9 | OTHER | NCS | Cytoplasmic |
| P0ABA8 | AtpG | 6 | 1 | 3.0 | 1.7 | 1.3 | 2.3 | 2.2 | 1.9 | OTHER | None | Cytoplasmic Membrane |
| A0A5Q2EJ51 | Z5646 | 12 | 5 | 3.6 | 1.6 | 0.7 | 2.7 | 2.9 | 0.9 | OTHER | None | Cytoplasmic |
| P0A8X3 | YceI | 19 | 9 | 0.3 | 1.6 | 1.0 | 1.6 | 2.9 | 2.2 | SPI | NCS | Unknown |
| **Reduced** | | | | | | | | | | | | |
| O82882 | StcE | 242 | 172 | -0.8 | -7.2 | -1.1 | 1.4 | 2.2 | 1.4 | SPI | NCS | Extracellular |
| P58319 | LtaE | 12 | 6 | -0.8 | -3.8 | -4.2 | 1.8 | 2.5 | 2.4 | OTHER | None | Cytoplasmic |
| P0A913 | Pal | 29 | 17 | -0.8 | -3.6 | -1.0 | 2.6 | 3.2 | 2.4 | SPII | NCS | Outer Membrane |
| P0ABB6 | AtpD | 16 | 8 | -1.3 | -3.0 | -1.5 | 2.6 | 2.9 | 2.3 | OTHER | None | Cytoplasmic Membrane |
| A0A5Q2EPR3 | CpdB | 41 | 20 | -0.5 | -3.0 | -0.4 | 2.7 | 3.7 | 1.2 | SPI | NCS | Periplasmic |
| P58694 | AsnS | 3 | 1 | -1.1 | -2.9 | -2.9 | 1.6 | 2.1 | 1.8 | OTHER | None | Cytoplasmic |
| A0A5B7NSE2 | EtpC | 3 | 1 | 0.7 | -2.4 | 0.7 | 2.6 | 3.4 | 1.7 | OTHER | None | Cytoplasmic Membrane |
| A0A4P8BAV1 | Z1498 | 33 | 18 | 0.1 | -2.3 | -0.2 | 0.7 | 3.0 | 0.9 | SPI | None | Unknown |
| P0A6N6 | Efp | 4 | 3 | -0.6 | -2.3 | -2.1 | 1.9 | 2.9 | 2.2 | OTHER | NCS | Cytoplasmic |
| A0A5Q2EU73 | PotD | 41 | 24 | -1.2 | -2.2 | -1.1 | 3.0 | 3.2 | 2.4 | SPI | NCS | Periplasmic |

**Table S6. Top proteins with highest *Q-value* in EHEC ΔT2SS in the absence of cells**

| **Accesion** | **Name** | **Peptide count** | **Unique peptides** | **Fold Change (Log2)** | | | ***Q value* (-Log10)** | | | **SignalP** | **SecretomeP** | **PSORTb** |
| --- | --- | --- | --- | --- | --- | --- | --- | --- | --- | --- | --- | --- |
|  |  |  |  | **ΔT1SS** | **ΔT2SS** | **ΔT3SS** | **ΔT1SS** | **ΔT2SS** | **ΔT3SS** |  |  |  |
| **Increased** | | | | | | | | | | | | |
| A0A4P8B4J6 | Z5009 | 8 | 4 | 0.7 | 2.5 | 0.8 | 1.3 | 3.2 | 0.8 | OTHER | None | Cytoplasmic |
| A0A5Q2ETC9 | VacJ | 8 | 4 | 2.6 | 1.4 | 1.3 | 2.9 | 3.2 | 1.5 | SPII | NCS | Outer Membrane |
| P62553 | CcdA | 1 | 1 | 1.3 | 1.2 | 0.8 | 3.2 | 3.2 | 2.4 | OTHER | None | Unknown |
| P67911 | HldD | 21 | 12 | 2.2 | 1.1 | -0.1 | 3.9 | 3.2 | 0.3 | OTHER | None | Cytoplasmic |
| P0A7S1 | RpsK | 16 | 11 | 0.1 | 1.7 | -0.6 | 0.4 | 3.0 | 0.9 | OTHER | NCS | Cytoplasmic |
| A0A6M7GZR0 | Z2206 | 6 | 4 | 1.1 | 1.5 | 0.7 | 2.6 | 3.0 | 0.8 | SPI | NCS | Unknown |
| P0AB08 | YcbK | 6 | 2 | 4.2 | 2.5 | 2.2 | 4.1 | 3.0 | 1.3 | SPI-Tat | None | Unknown |
| P0ABP9 | DeoD | 37 | 25 | 0.6 | 1.3 | 0.4 | 2.3 | 3.0 | 1.5 | OTHER | None | Cytoplasmic |
| P0A8X3 | YceI | 19 | 9 | 0.3 | 1.6 | 1.0 | 1.6 | 2.9 | 2.2 | SPI | NCS | Unknown |
| A0A5Q2EJ51 | Z5646 | 12 | 5 | 3.6 | 1.6 | 0.7 | 2.7 | 2.9 | 0.9 | OTHER | None | Cytoplasmic |
| **Reduced** | | | | | | | | | | | | |
| A0A5Q2EPR3 | CpdB | 41 | 20 | -0.5 | -3.0 | -0.4 | 2.7 | 3.7 | 1.2 | SPI | NCS | Periplasmic |
| A0A6M7GWT5 | MglB | 50 | 34 | -0.7 | -1.6 | -1.9 | 2.1 | 3.7 | 2.7 | SPI | NCS | Periplasmic |
| A0A5B7NSE2 | EtpC | 3 | 1 | 0.7 | -2.4 | 0.7 | 2.6 | 3.4 | 1.7 | OTHER | None | Cytoplasmic Membrane |
| A0A6M7H272 | PepD | 33 | 13 | -0.5 | -1.3 | -2.8 | 1.5 | 3.2 | 2.5 | OTHER | None | Cytoplasmic |
| P0A913 | Pal | 29 | 17 | -0.8 | -3.6 | -1.0 | 2.6 | 3.2 | 2.4 | SPII | NCS | Outer Membrane |
| A0A5Q2EU73 | PotD | 41 | 24 | -1.2 | -2.2 | -1.1 | 3.0 | 3.2 | 2.4 | SPI | NCS | Periplasmic |
| Q7BSW5 | EspP | 274 | 193 | -1.3 | -1.0 | -1.2 | 3.0 | 3.2 | 2.3 | SPI | NCS | Outer Membrane |
| A0A4P8BAV1 | Z1498 | 33 | 18 | 0.1 | -2.3 | -0.2 | 0.7 | 3.0 | 0.9 | SPI | None | Unknown |
| A0A6M7GT08 | EspB | 159 | 120 | -0.6 | -2.1 | -5.2 | 2.1 | 2.9 | 2.7 | OTHER | NCS | Extracellular |
| P0A838 | SucC | 19 | 12 | -0.8 | -1.8 | -1.0 | 2.5 | 2.9 | 2.3 | OTHER | None | Cytoplasmic |

**Table S7. Top ten proteins with highest fold change in EHEC ΔT2SS in the presence of cells**

| **Accesion** | **Name** | **Peptide count** | **Unique peptides** | **Fold Change (Log2)** | | | ***Q value* (-Log10)** | | | **SignalP** | **SecretomeP** | **PSORTb** |
| --- | --- | --- | --- | --- | --- | --- | --- | --- | --- | --- | --- | --- |
|  |  |  |  | **ΔT1SS** | **ΔT2SS** | **ΔT3SS** | **ΔT1SS** | **ΔT2SS** | **ΔT3SS** |  |  |  |
| **Increased** | | | | | | | | | | | | |
| A0A0H3JIJ0 | Z3275 | 3 | 3 | 0.3 | 1.4 | 0.1 | 0.5 | 2.1 | 0.2 | OTHER | None | Unknown |
| P0A7M4 | RpmB | 2 | 1 | 0.1 | 1.3 | 0.3 | 0.1 | 2.3 | 0.5 | OTHER | NCS | Cytoplasmic |
| P0A6P3 | Tsf | 30 | 27 | 0.2 | 1.1 | 0.2 | 0.4 | 2.5 | 0.5 | OTHER | None | Cytoplasmic |
| P0AF91 | RraB | 5 | 3 | 0.1 | 1.1 | -2.9 | 0.1 | 2.1 | 1.9 | OTHER | None | Cytoplasmic |
| P0A7T5 | RpsP | 8 | 8 | 0.1 | 1.1 | -0.3 | 0.1 | 2.3 | 1.1 | OTHER | None | Cytoplasmic |
| P58217 | Z2083 | 4 | 3 | 0.2 | 1.1 | -1.0 | 0.3 | 2.0 | 1.3 | OTHER | NCS | Unknown |
| **Reduced** | | | | | | | | | | | | |
| O82882 | StcE | 168 | 154 | -0.2 | -6.7 | 0.5 | 0.5 | 3.0 | 1.7 | SPI | NCS | Extracellular |
| P0A913 | Pal | 15 | 13 | 0.4 | -3.8 | 0.4 | 1.1 | 4.8 | 0.4 | SPII | NCS | Outer Membrane |
| A0A5Q2EPV8 | Z5884 | 3 | 3 | -0.1 | -2.6 | 0.2 | 0.2 | 2.1 | 0.2 | OTHER | NCS | Unknown |
| A0A6M7H7A3 | Z0372 | 1 | 1 | 0.0 | -2.3 | 2.3 | 0.0 | 2.4 | 3.5 | SPI | None | Unknown |
| A0A0H3JKM0 | YhcN | 4 | 3 | -0.3 | -1.8 | -0.3 | 0.7 | 2.3 | 0.9 | SPI | NCS | Unknown |
| A0A0H3JL10 | Slp | 1 | 1 | -0.4 | -1.6 | 0.8 | 0.9 | 2.3 | 1.8 | SPII | NCS | Outer Membrane |
| A0A6M6W7P0 | BorW | 5 | 3 | 0.9 | -1.5 | 0.1 | 1.1 | 2.0 | 0.3 | SPII | NCS | Unknown |
| P0A9Y1 | CspA | 6 | 6 | 0.1 | -1.5 | 0.9 | 0.2 | 2.4 | 2.4 | OTHER | NCS | Cytoplasmic |
| Q8X9M2 | RpsO | 6 | 4 | 0.3 | -1.5 | 1.2 | 1.1 | 2.4 | 1.9 | OTHER | None | Cytoplasmic |
| Q8XB75 | HiuH | 3 | 2 | 0.4 | -1.5 | 0.5 | 1.1 | 2.4 | 2.1 | SPI | NCS | Unknown |
| Q8X3U3 | YehC | 4 | 3 | -0.3 | -1.5 | -1.6 | 0.7 | 2.4 | 2.5 | SPI | NCS | Periplasmic |
| A0A4P8BAU8 | Stx2B | 13 | 13 | 0.4 | -1.4 | 1.0 | 0.9 | 3.0 | 2.4 | SPI | NCS | Periplasmic |
| A0A4P8B4A7 | EspD | 68 | 61 | 0.3 | -1.3 | -2.7 | 0.7 | 2.5 | 3.0 | OTHER | NCS | Extracellular |
| Q7BSW5 | EspP | 304 | 275 | 0.0 | -1.3 | 0.0 | 0.0 | 2.9 | 0.3 | SPI | NCS | Outer Membrane |

**Table S8. Top ten proteins with highest *Q-value* in EHEC ΔT2SS in the presence of cells**

| **Accesion** | **Name** | **Peptide count** | **Unique peptides** | **Fold Change (Log2)** | | | ***Q value* (-Log10)** | | | **SignalP** | **SecretomeP** | **PSORTb** |
| --- | --- | --- | --- | --- | --- | --- | --- | --- | --- | --- | --- | --- |
|  |  |  |  | **ΔT1SS** | **ΔT2SS** | **ΔT3SS** | **ΔT1SS** | **ΔT2SS** | **ΔT3SS** |  |  |  |
| **Increased** | | | | | | | | | | | | |
| P0A6P3 | Tsf | 30 | 27 | 0.2 | 1.1 | 0.2 | 0.4 | 2.5 | 0.5 | OTHER | None | Cytoplasmic |
| P0A7T5 | RpsP | 8 | 8 | 0.1 | 1.1 | -0.3 | 0.1 | 2.3 | 1.1 | OTHER | None | Cytoplasmic |
| P0A7M4 | RpmB | 2 | 1 | 0.1 | 1.3 | 0.3 | 0.1 | 2.3 | 0.5 | OTHER | NCS | Cytoplasmic |
| P0AF91 | RraB | 5 | 3 | 0.1 | 1.1 | -2.9 | 0.1 | 2.1 | 1.9 | OTHER | None | Cytoplasmic |
| A0A0H3JIJ0 | Z3275 | 3 | 3 | 0.3 | 1.4 | 0.1 | 0.5 | 2.1 | 0.2 | OTHER | None | Unknown |
| P58217 | Z2083 | 4 | 3 | 0.2 | 1.1 | -1.0 | 0.3 | 2.0 | 1.3 | OTHER | NCS | Unknown |
| **Reduced** | | | | | | | | | | | | |
| P0A913 | Pal | 15 | 13 | 0.4 | -3.8 | 0.4 | 1.1 | 4.8 | 0.4 | SPII | NCS | Outer Membrane |
| A0A4P8BAU8 | Stx2B | 13 | 13 | 0.4 | -1.4 | 1.0 | 0.9 | 3.0 | 2.4 | SPI | NCS | Periplasmic |
| O82882 | StcE | 168 | 154 | -0.2 | -6.7 | 0.5 | 0.5 | 3.0 | 1.7 | SPI | NCS | Extracellular |
| P0A6F7 | GroEL | 59 | 53 | -1.1 | -1.2 | -0.7 | 1.4 | 2.9 | 2.7 | OTHER | None | Cytoplasmic |
| Q7BSW5 | EspP | 304 | 275 | 0.0 | -1.3 | 0.0 | 0.0 | 2.9 | 0.3 | SPI | NCS | Outer Membrane |
| P0A9B4 | GapA | 84 | 72 | -0.2 | -1.1 | 0.2 | 0.5 | 2.5 | 1.1 | OTHER | NCS | Cytoplasmic |
| P66607 | RpsG | 30 | 28 | 0.3 | -1.2 | 1.2 | 0.8 | 2.5 | 2.5 | OTHER | None | Cytoplasmic |
| A0A4P8B4A7 | EspD | 68 | 61 | 0.3 | -1.3 | -2.7 | 0.7 | 2.5 | 3.0 | OTHER | NCS | Extracellular |
| P60440 | RplC | 18 | 16 | 0.0 | -1.1 | 0.8 | 0.0 | 2.4 | 2.7 | OTHER | NCS | Cytoplasmic |
| Q8X9M2 | RpsO | 6 | 4 | 0.3 | -1.5 | 1.2 | 1.1 | 2.4 | 1.9 | OTHER | None | Cytoplasmic |
| P0A6B0 | AcpP | 21 | 19 | 0.2 | -1.2 | 1.8 | 0.5 | 2.4 | 3.0 | OTHER | None | Cytoplasmic |
| P0ABA5 | AtpH | 6 | 6 | -0.2 | -1.1 | -0.6 | 0.6 | 2.4 | 1.6 | OTHER | None | Cytoplasmic |
| Q8XB75 | HiuH | 3 | 2 | 0.4 | -1.5 | 0.5 | 1.1 | 2.4 | 2.1 | SPI | NCS | Unknown |
| P0A9Y1 | CspA | 6 | 6 | 0.1 | -1.5 | 0.9 | 0.2 | 2.4 | 2.4 | OTHER | NCS | Cytoplasmic |

**Table S9. Top ten proteins with highest fold change in EHEC ΔT3SS in the absence of cells**

| **Accesion** | **Name** | **Peptide count** | **Unique peptides** | **Fold Change (Log2)** | | | ***Q value* (-Log10)** | | | **SignalP** | **SecretomeP** | **PSORTb** |
| --- | --- | --- | --- | --- | --- | --- | --- | --- | --- | --- | --- | --- |
|  |  |  |  | **ΔT1SS** | **ΔT2SS** | **ΔT3SS** | **ΔT1SS** | **ΔT2SS** | **ΔT3SS** |  |  |  |
| **Increased** | | | | | | | | | | | | |
| P0AAD0 | IscA | 3 | 1 | 4.6 | 3.5 | 2.2 | 3.0 | 2.0 | 2.1 | OTHER | NCS | Cytoplasmic |
| P63624 | AsnA | 3 | 2 | 1.5 | 0.9 | 2.1 | 2.6 | 1.8 | 2.7 | OTHER | None | Cytoplasmic |
| P0AAI7 | FabF | 7 | 5 | -0.5 | -0.4 | 1.6 | 0.8 | 0.6 | 2.4 | OTHER | None | Cytoplasmic |
| A0A0H3JHG0 | NlpB | 19 | 15 | 1.4 | 1.2 | 1.3 | 2.9 | 1.9 | 2.4 | SPII | NCS | Outer Membrane |
| Q8X3W0 | Z0982 | 31 | 6 | 0.7 | 0.8 | 1.0 | 2.6 | 2.9 | 2.2 | OTHER | NCS | Extracellular |
| P0A8X3 | YceI | 19 | 9 | 0.3 | 1.6 | 1.0 | 1.6 | 2.9 | 2.2 | SPI | NCS | Unknown |
| A0A6M7GXB3 | YrfE | 2 | 2 | -0.6 | 0.7 | 1.0 | 1.2 | 1.5 | 2.0 | OTHER | None | Cytoplasmic |
| **Reduced** | | | | | | | | | | | | |
| A0A6M7GT08 | EspB | 159 | 120 | -0.6 | -2.1 | -5.2 | 2.1 | 2.9 | 2.7 | OTHER | NCS | Extracellular |
| A0A4P8B4A7 | EspD | 55 | 46 | -0.2 | -0.8 | -4.5 | 1.2 | 2.1 | 2.6 | OTHER | NCS | Extracellular |
| P58319 | LtaE | 12 | 6 | -0.8 | -3.8 | -4.2 | 1.8 | 2.5 | 2.4 | OTHER | None | Cytoplasmic |
| A0A6M7GSY4 | Map | 9 | 4 | -0.5 | -1.1 | -3.9 | 1.6 | 2.0 | 2.4 | OTHER | NCS | Unknown |
| A0A5Q2EW70 | EspA | 48 | 38 | -0.7 | -0.9 | -3.8 | 1.8 | 1.9 | 2.4 | OTHER | NCS | Unknown |
| P0AEK5 | FabI | 11 | 6 | -1.2 | -1.2 | -3.6 | 2.0 | 1.9 | 2.3 | OTHER | None | Cytoplasmic Membrane |
| A0A4P8BDD0 | Z1432 | 3 | 2 | -1.3 | -0.5 | -2.9 | 2.6 | 1.1 | 2.5 | OTHER | None | Cytoplasmic |
| A0A6M7H272 | PepD | 33 | 13 | -0.5 | -1.3 | -2.8 | 1.5 | 3.2 | 2.5 | OTHER | None | Cytoplasmic |
| P68067 | GrcA | 10 | 6 | -1.5 | -2.0 | -2.5 | 2.5 | 2.2 | 2.3 | OTHER | None | Cytoplasmic |
| P0A736 | MinE | 1 | 1 | -1.4 | -1.6 | -2.3 | 2.5 | 2.3 | 2.4 | OTHER | None | Cytoplasmic |
| P0A9L1 | SlyD | 1 | 1 | -0.4 | -0.6 | -2.2 | 2.5 | 2.2 | 2.7 | OTHER | None | Cytoplasmic |
| P0A6N6 | Efp | 4 | 3 | -0.6 | -2.3 | -2.1 | 1.9 | 2.9 | 2.2 | OTHER | NCS | Cytoplasmic |
| P58209 | DapB | 19 | 10 | -0.1 | -0.9 | -2.0 | 1.4 | 3.7 | 3.0 | OTHER | None | Cytoplasmic |

**Table S10. Top ten proteins with highest *Q-value* in EHEC ΔT3SS in the absence of cells**

| **Accesion** | **Name** | **Peptide count** | **Unique peptides** | **Fold Change (Log2)** | | | ***Q value* (-Log10)** | | | **SignalP** | **SecretomeP** | **PSORTb** |
| --- | --- | --- | --- | --- | --- | --- | --- | --- | --- | --- | --- | --- |
|  |  |  |  | **ΔT1SS** | **ΔT2SS** | **ΔT3SS** | **ΔT1SS** | **ΔT2SS** | **ΔT3SS** |  |  |  |
| **Increased** | | | | | | | | | | | | |
| P63624 | AsnA | 3 | 2 | 1.5 | 0.9 | 2.1 | 2.6 | 1.8 | 2.7 | OTHER | None | Cytoplasmic |
| A0A0H3JHG0 | NlpB | 19 | 15 | 1.4 | 1.2 | 1.3 | 2.9 | 1.9 | 2.4 | SPII | NCS | Outer Membrane |
| P0AAI7 | FabF | 7 | 5 | -0.5 | -0.4 | 1.6 | 0.8 | 0.6 | 2.4 | OTHER | None | Cytoplasmic |
| Q8X3W0 | Z0982 | 31 | 6 | 0.7 | 0.8 | 1.0 | 2.6 | 2.9 | 2.2 | OTHER | NCS | Extracellular |
| P0A8X3 | YceI | 19 | 9 | 0.3 | 1.6 | 1.0 | 1.6 | 2.9 | 2.2 | SPI | NCS | Unknown |
| P0AAD0 | IscA | 3 | 1 | 4.6 | 3.5 | 2.2 | 3.0 | 2.0 | 2.1 | OTHER | NCS | Cytoplasmic |
| A0A6M7GXB3 | YrfE | 2 | 2 | -0.6 | 0.7 | 1.0 | 1.2 | 1.5 | 2.0 | OTHER | None | Cytoplasmic |
| **Reduced** | | | | | | | | | | | | |
| P58209 | DapB | 19 | 10 | -0.1 | -0.9 | -2.0 | 1.4 | 3.7 | 3.0 | OTHER | None | Cytoplasmic |
| P0A9L1 | SlyD | 1 | 1 | -0.4 | -0.6 | -2.2 | 2.5 | 2.2 | 2.7 | OTHER | None | Cytoplasmic |
| A0A6M7GT08 | EspB | 159 | 120 | -0.6 | -2.1 | -5.2 | 2.1 | 2.9 | 2.7 | OTHER | NCS | Extracellular |
| A0A6M7GWT5 | MglB | 50 | 34 | -0.7 | -1.6 | -1.9 | 2.1 | 3.7 | 2.7 | SPI | NCS | Periplasmic |
| A0A4P8B4A7 | EspD | 55 | 46 | -0.2 | -0.8 | -4.5 | 1.2 | 2.1 | 2.6 | OTHER | NCS | Extracellular |
| A0A6M7H272 | PepD | 33 | 13 | -0.5 | -1.3 | -2.8 | 1.5 | 3.2 | 2.5 | OTHER | None | Cytoplasmic |
| A0A4P8BDD0 | Z1432 | 3 | 2 | -1.3 | -0.5 | -2.9 | 2.6 | 1.1 | 2.5 | OTHER | None | Cytoplasmic |
| P0A736 | MinE | 1 | 1 | -1.4 | -1.6 | -2.3 | 2.5 | 2.3 | 2.4 | OTHER | None | Cytoplasmic |
| Q8XE30 | GyrA | 7 | 3 | -0.9 | 0.3 | -1.7 | 2.5 | 0.4 | 2.4 | OTHER | None | Cytoplasmic |
| A0A5Q2EU73 | PotD | 41 | 24 | -1.2 | -2.2 | -1.1 | 3.0 | 3.2 | 2.4 | SPI | NCS | Periplasmic |
| A0A6M7GSY4 | Map | 9 | 4 | -0.5 | -1.1 | -3.9 | 1.6 | 2.0 | 2.4 | OTHER | NCS | Unknown |
| A0A5Q2EW70 | EspA | 48 | 38 | -0.7 | -0.9 | -3.8 | 1.8 | 1.9 | 2.4 | OTHER | NCS | Unknown |
| P58319 | LtaE | 12 | 6 | -0.8 | -3.8 | -4.2 | 1.8 | 2.5 | 2.4 | OTHER | None | Cytoplasmic |

**Table S11. Top ten proteins with highest fold change in EHEC ΔT3SS in the presence of cells**

| **Accesion** | **Name** | **Peptide count** | **Unique peptides** | **Fold Change (Log2)** | | | ***Q value* (-Log10)** | | | **SignalP** | **SecretomeP** | **PSORTb** |
| --- | --- | --- | --- | --- | --- | --- | --- | --- | --- | --- | --- | --- |
|  |  |  |  | **ΔT1SS** | **ΔT2SS** | **ΔT3SS** | **ΔT1SS** | **ΔT2SS** | **ΔT3SS** |  |  |  |
| **Increased** | | | | | | | | | | | | |
| P0AD51 | YfiA | 11 | 9 | 0.3 | 0.1 | 2.8 | 0.5 | 0.2 | 3.0 | OTHER | None | Cytoplasmic |
| A0A6M6W3H8 | Z1430 | 5 | 3 | 1.1 | 0.3 | 2.7 | 0.8 | 0.3 | 2.4 | OTHER | NCS | Unknown |
| A0A6M7H7A3 | Z0372 | 1 | 1 | 0.0 | -2.3 | 2.3 | 0.0 | 2.4 | 3.5 | SPI | None | Unknown |
| A0A4P8B779 | YeaF | 13 | 11 | 0.6 | 0.4 | 2.2 | 1.1 | 0.7 | 3.0 | SPI | NCS | Outer Membrane |
| A0A6M7GSJ8 | AnsB | 12 | 9 | 0.9 | 0.9 | 2.0 | 0.3 | 0.4 | 2.5 | SPI | NCS | Periplasmic |
| P0A6B0 | AcpP | 21 | 19 | 0.2 | -1.2 | 1.8 | 0.5 | 2.4 | 3.0 | OTHER | None | Cytoplasmic |
| P0A9B1 | Fur | 2 | 2 | 0.6 | -0.6 | 1.8 | 1.2 | 1.7 | 3.0 | OTHER | None | Cytoplasmic |
| A0A4P8B947 | Z2967 | 3 | 2 | 0.1 | -0.5 | 1.7 | 0.6 | 0.6 | 2.8 | SPI | NCS | Unknown |
| P0ACZ1 | YebG | 1 | 1 | 0.5 | -1.9 | 1.7 | 0.6 | 1.8 | 2.1 | OTHER | NCS | Unknown |
| A0A6M7GYX7 | BetW | 4 | 2 | -0.5 | 2.2 | 1.7 | 0.2 | 0.6 | 2.2 | OTHER | None | Unknown |
| **Reduced** | | | | | | | | | | | | |
| A0A6M7GT08 | EspB | 142 | 125 | 0.5 | -1.3 | -4.6 | 0.7 | 2.3 | 2.9 | OTHER | NCS | Extracellular |
| P0A871 | TalB | 22 | 19 | 0.3 | -1.1 | -3.9 | 0.3 | 1.6 | 2.3 | OTHER | None | Cytoplasmic |
| Q8X5A7 | Z3074 | 35 | 5 | -0.8 | -2.2 | -3.5 | 0.8 | 1.9 | 2.1 | OTHER | NCS | Extracellular |
| P0AEM2 | FkpB | 2 | 1 | -1.1 | -1.0 | -3.2 | 1.2 | 2.0 | 3.0 | OTHER | None | Cytoplasmic |
| A0A4P8B4A7 | EspD | 68 | 61 | 0.3 | -1.3 | -2.7 | 0.7 | 2.5 | 3.0 | OTHER | NCS | Extracellular |
| P65765 | FkpA | 20 | 18 | -0.4 | -1.3 | -2.6 | 0.6 | 2.0 | 2.4 | SPI | NCS | Periplasmic |
| A0A6M7H1Z5 | PflB | 98 | 83 | 0.2 | -1.1 | -2.3 | 0.3 | 1.7 | 2.3 | OTHER | None | Cytoplasmic |
| P0AG69 | RpsA | 52 | 46 | 0.1 | -0.8 | -1.7 | 0.1 | 2.0 | 2.9 | OTHER | None | Cytoplasmic |
| Q8X3U3 | YehC | 4 | 3 | -0.3 | -1.5 | -1.6 | 0.7 | 2.4 | 2.5 | SPI | NCS | Periplasmic |
| P0A957 | Eda | 3 | 3 | -0.4 | -1.0 | -1.5 | 1.1 | 1.7 | 2.9 | OTHER | None | Cytoplasmic |

**Table S12. Top ten proteins with highest *Q-value* in EHEC ΔT3SS in the presence of cells**

| **Accesion** | **Name** | **Peptide count** | **Unique peptides** | **Fold Change (Log2)** | | | ***Q value* (-Log10)** | | | **SignalP** | **SecretomeP** | **PSORTb** |
| --- | --- | --- | --- | --- | --- | --- | --- | --- | --- | --- | --- | --- |
|  |  |  |  | **ΔT1SS** | **ΔT2SS** | **ΔT3SS** | **ΔT1SS** | **ΔT2SS** | **ΔT3SS** |  |  |  |
| **Increased** | | | | | | | | | | | | |
| A0A6M7H7A3 | Z0372 | 1 | 1 | 0.0 | -2.3 | 2.3 | 0.0 | 2.4 | 3.5 | SPI | None | Unknown |
| P0A9Y8 | CspC | 13 | 12 | 0.1 | -0.1 | 1.1 | 0.4 | 0.7 | 3.0 | OTHER | NCS | Cytoplasmic |
| P0AD51 | YfiA | 11 | 9 | 0.3 | 0.1 | 2.8 | 0.5 | 0.2 | 3.0 | OTHER | None | Cytoplasmic |
| P0AG53 | RpmD | 4 | 3 | 0.2 | -0.7 | 1.1 | 0.8 | 1.2 | 3.0 | OTHER | None | Cytoplasmic |
| P62401 | RplE | 20 | 18 | 0.0 | -0.4 | 1.2 | 0.1 | 1.0 | 3.0 | OTHER | None | Cytoplasmic |
| P0A6B0 | AcpP | 21 | 19 | 0.2 | -1.2 | 1.8 | 0.5 | 2.4 | 3.0 | OTHER | None | Cytoplasmic |
| P0A9B1 | Fur | 2 | 2 | 0.6 | -0.6 | 1.8 | 1.2 | 1.7 | 3.0 | OTHER | None | Cytoplasmic |
| Q7DBI0 | FliC | 125 | 110 | -0.1 | 0.0 | 1.5 | 0.2 | 0.2 | 3.0 | OTHER | NCS | Extracellular |
| A0A4P8B779 | YeaF | 13 | 11 | 0.6 | 0.4 | 2.2 | 1.1 | 0.7 | 3.0 | SPI | NCS | Outer Membrane |
| A0A6M6S3U5 | Z2099 | 12 | 2 | 0.3 | -0.1 | 1.4 | 0.7 | 0.4 | 3.0 | SPI | NCS | Unknown |
| **Reduced** | | | | | | | | | | | | |
| P0AEM2 | FkpB | 2 | 1 | -1.1 | -1.0 | -3.2 | 1.2 | 2.0 | 3.0 | OTHER | None | Cytoplasmic |
| A0A4P8B4A7 | EspD | 68 | 61 | 0.3 | -1.3 | -2.7 | 0.7 | 2.5 | 3.0 | OTHER | NCS | Extracellular |
| P0A957 | Eda | 3 | 3 | -0.4 | -1.0 | -1.5 | 1.1 | 1.7 | 2.9 | OTHER | None | Cytoplasmic |
| P0AG69 | RpsA | 52 | 46 | 0.1 | -0.8 | -1.7 | 0.1 | 2.0 | 2.9 | OTHER | None | Cytoplasmic |
| A0A6M7GT08 | EspB | 142 | 125 | 0.5 | -1.3 | -4.6 | 0.7 | 2.3 | 2.9 | OTHER | NCS | Extracellular |
| Q8X3U3 | YehC | 4 | 3 | -0.3 | -1.5 | -1.6 | 0.7 | 2.4 | 2.5 | SPI | NCS | Periplasmic |
| P61951 | FldA | 2 | 2 | 0.0 | 1.1 | -1.5 | 0.0 | 0.6 | 2.4 | OTHER | None | Cytoplasmic |
| P65765 | FkpA | 20 | 18 | -0.4 | -1.3 | -2.6 | 0.6 | 2.0 | 2.4 | SPI | NCS | Periplasmic |
| P0A871 | TalB | 22 | 19 | 0.3 | -1.1 | -3.9 | 0.3 | 1.6 | 2.3 | OTHER | None | Cytoplasmic |
| A0A6M7H1Z5 | PflB | 98 | 83 | 0.2 | -1.1 | -2.3 | 0.3 | 1.7 | 2.3 | OTHER | None | Cytoplasmic |

**Table S13. List of possible new secreted substrates due to selected features**

| **ID** | **Name** | **SignalP** | **SecretomeP** | **PSORTb** | **Secretion System**  **(SS)** |  | **BastionHub Prediction** | | | | | | **Absent in** |
| --- | --- | --- | --- | --- | --- | --- | --- | --- | --- | --- | --- | --- | --- |
|  |  |  |  |  |  | **Length**  **(aa)** | **T1SS** | **T2SS** | **T3SS** | **T4SS** | **T6SS** | **Possible SS** |  |
| **Proteins identified in supernatants without cells** | | | | | | | | | | | | | |
| Q8XE47 | NapA | SPI-Tat | NCS | Periplasmic | Unknown | 828 | 0.048 | 0.224 | 0.015 | 0.024 | 0.049 | '- | ΔT1/T2/T3SS |
| A0A0H3JHG0 | NlpB | SPII | NCS | Outer Membrane | Unknown | 345 | 0.08 | 0.534* | 0.09 | 0.188 | 0.06 | II | ΔT1SS |
| A0A6M7GXW6 | YddB | SPI | NCS | Outer Membrane | Unknown | 790 | 0.263 | 0.802* | 0.103 | 0.1 | 0.19 | II | ΔT1SS |
| P61321 | LolB | SPII | NCS | Periplasmic | Unknown | 207 | 0.074 | 0.389 | 0.066 | 0.092 | 0.052 | '- | ΔT1SS |
| A0A5Q2EPE0 | AfuA | SPI | NCS | Periplasmic | Unknown | 343 | 0.058 | 0.564* | 0.022 | 0.041 | 0.019 | II | ΔT1SS |
| P0AB39 | LpoB | SPII | NCS | Unknown | Unknown | 213 | 0.068 | 0.574* | 0.075 | 0.059 | 0.069 | II | ΔT1SS |
| Q8X6N7 | YbjP | SPII | NCS | Unknown | Unknown | 173 | 0.16 | 0.937* | 0.251 | 0.179 | 0.101 | II | ΔT1SS |
| A0A6M7H3V5 | Z2887 | SPI | NCS | Unknown | Unknown | 113 | 0.1 | 0.983* | 0.056 | 0.25 | 0.054 | II | ΔT1SS |
| A0A6M7H4A0 | PhoE | SPI | NCS | Outer Membrane | Unknown | 351 | 0.324 | 0.279 | 0.189 | 0.112 | 0.207 | '- | ΔT3SS |
| Q8XDZ4 | Spy | SPI | NCS | Periplasmic | Unknown | 161 | 0.225 | 0.198 | 0.271 | 0.111 | 0.103 | '- | ΔT3SS |
| A0A6M0JSH9 | Z3091 | OTHER | NCS | Unknown | Unknown | 108 | 0.345 | 0.219 | 0.256 | 0.143 | 0.321 | '- | ΔT3SS |
| A0A6M7GW74 | NlpD | SPII | NCS | Outer Membrane | Unknown | 379 | 0.062 | 0.296 | 0.042 | 0.063 | 0.034 | '- | ΔT1/T2SS |
| Q8X5U3 | NikA | SPI | None | Periplasmic | Unknown | 524 | 0.094 | 0.629* | 0.049 | 0.034 | 0.033 | II | ΔT1/T2SS |
| A0A4P8B641 | Z3087 | OTHER | NCS | Unknown | Unknown | 250 | 0.293 | 0.352 | 0.33 | 0.24 | 0.846 | VI | ΔT1/T3SS |
| Q8X3X8 | Z1793 | OTHER | NCS | Unknown | Unknown | 617 | 0.2 | 0.687* | 0.112 | 0.071 | 0.27 | II | ΔT1/T3SS |
| **Proteins identified in supernatants with cells** | | | | | | | | | | | | | |
| Q8X4C8 | Z1382 | OTHER | NCS | Extracellular | Unknown | 375 | 0.339 | 0.236 | 0.208 | 0.114 | 0.236 | '- | ΔT1/T2/T3SS |
| Q8X9Q0 | Z2334 | OTHER | NCS | Outer Membrane | Unknown | 255 | 0.35 | 0.389 | 0.17 | 0.095 | 0.197 | '- | ΔT1/T2/T3SS |
| A0A5Q2EU18 | Z6027 | OTHER | NCS | Extracellular | Unknown | 437 | 0.382 | 0.293 | 0.241 | 0.129 | 0.309 | '- | ΔT1SS |
| Q8X3W0 | Z0982 | OTHER | NCS | Extracellular | Unknown | 440 | 0.401 | 0.311 | 0.252 | 0.137 | 0.352 | '- | ΔT1SS |
| Q8X5B4 | Z2340 | OTHER | NCS | Extracellular | Unknown | 439 | 0.397 | 0.317 | 0.261 | 0.134 | 0.336 | '- | ΔT1SS |
| A0A4P8B8F8 | Z2239 | SPI | NCS | Outer Membrane | Unknown | 366 | 0.306 | 0.358 | 0.156 | 0.097 | 0.176 | '- | ΔT1SS |
| A0A6M7H1K1 | ModA | SPI | NCS | Periplasmic | Unknown | 257 | 0.087 | 0.146 | 0.05 | 0.051 | 0.016 | '- | ΔT1SS |
| A0A5Q2EU73 | PotD | SPI | NCS | Periplasmic | Unknown | 348 | 0.037 | 0.32 | 0.02 | 0.03 | 0.006 | '- | ΔT1SS |
| P0AEY0 | MalE | SPI | NCS | Periplasmic | Unknown | 396 | 0.046 | 0.302 | 0.021 | 0.024 | 0.01 | '- | ΔT1SS |
| P0AEQ5 | GlnH | SPI | NCS | Periplasmic | Unknown | 248 | 0.097 | 0.15 | 0.053 | 0.057 | 0.025 | '- | ΔT1SS |
| Q8XDA4 | OppA | SPI | NCS | Periplasmic | Unknown | 543 | 0.056 | 0.151 | 0.031 | 0.045 | 0.014 | '- | ΔT1SS |
| P0C0V1 | DegP | SPI | NCS | Periplasmic | Unknown | 474 | 0.108 | 0.311 | 0.052 | 0.022 | 0.016 | '- | ΔT1SS |
| A0A6M7H470 | FliY | SPI | NCS | Periplasmic | Unknown | 266 | 0.086 | 0.174 | 0.057 | 0.053 | 0.024 | '- | ΔT1SS |
| P65765 | FkpA | SPI | NCS | Periplasmic | Unknown | 270 | 0.052 | 0.121 | 0.068 | 0.045 | 0.017 | '- | ΔT1SS |
| A0A4P8B8B9 | Z3342 | OTHER | NCS | Unknown | Unknown | 648 | 0.213 | 0.85* | 0.109 | 0.071 | 0.364 | II | ΔT1SS |
| A0A6M0JIT3 | Stx2A | SPI | None | Unknown | Stx | 319 | 0.604 | 0.77 | 0.934* | 0.884 | 0.874 | I II III IV VI | ΔT1SS |
| A0A6M6XA91 | Z1483 | OTHER | NCS | Unknown | Unknown | 645 | 0.513* | 0.538 | 0.315 | 0.162 | 0.343 | I II | ΔT1SS |
| Q8X2B4 | Z1466 | OTHER | NCS | Unknown | Unknown | 645 | 0.217 | 0.82* | 0.114 | 0.073 | 0.34 | II | ΔT1SS |
| P0AGD5 | SodB | OTHER | NCS | Periplasmic | Unknown | 193 | 0.196 | 0.113 | 0.904* | 0.09 | 0.076 | III | ΔT1/T2SS |
| P0A913 | Pal | SPII | NCS | Outer Membrane | Unknown | 173 | 0.079 | 0.133 | 0.06 | 0.057 | 0.026 | '- | ΔT1/T2SS |

*The highest prediction scores for the secretion systems.
